# miR-146a-5p mediates atherogenic signalling from immune to vascular cells

**DOI:** 10.1093/cvr/cvag075

**Published:** 2026-04-13

**Authors:** Montserrat Climent, Stefania Zani, Nicolò Salvarani, Marco Cremonesi, Simone Serio, Anna Sbalchiero, Alfonso Tramontano, Luca Lambroia, Alice Mallia, Efrem Civilini, Cristina Banfi, Leonardo Elia

**Affiliations:** Department of Cardiovascular Medicine, IRCCS Humanitas Research Hospital, Rozzano, Milan 20089, Italy; Department of Biomedical Sciences, Humanitas University, Pieve Emanuele, Milan 20072, Italy; Department of Cardiovascular Medicine, IRCCS Humanitas Research Hospital, Rozzano, Milan 20089, Italy; Department of Cardiovascular Medicine, IRCCS Humanitas Research Hospital, Rozzano, Milan 20089, Italy; Institute of Genetics and Biomedical Research, UoS Milan, National Research Council, Rozzano, Milan 20089, Italy; Department of Cardiovascular Medicine, IRCCS Humanitas Research Hospital, Rozzano, Milan 20089, Italy; Department of Cardiovascular Medicine, IRCCS Humanitas Research Hospital, Rozzano, Milan 20089, Italy; Institute of Genetics and Biomedical Research, UoS Milan, National Research Council, Rozzano, Milan 20089, Italy; Department of Cardiovascular Medicine, IRCCS Humanitas Research Hospital, Rozzano, Milan 20089, Italy; Department of Biomedical Sciences, Humanitas University, Pieve Emanuele, Milan 20072, Italy; Department of Cardiovascular Medicine, IRCCS Humanitas Research Hospital, Rozzano, Milan 20089, Italy; Department of Molecular and Translational Medicine, University of Brescia, Brescia 25123, Italy; Department of Cardiovascular Medicine, IRCCS Humanitas Research Hospital, Rozzano, Milan 20089, Italy; Unit of Functional Proteomics, Metabolomics and Network Analysis, Centro Cardiologico Monzino IRCCS, Milan 20138, Italy; Department of Cardiovascular Medicine, IRCCS Humanitas Research Hospital, Rozzano, Milan 20089, Italy; Department of Biomedical Sciences, Humanitas University, Pieve Emanuele, Milan 20072, Italy; Unit of Functional Proteomics, Metabolomics and Network Analysis, Centro Cardiologico Monzino IRCCS, Milan 20138, Italy; Department of Cardiovascular Medicine, IRCCS Humanitas Research Hospital, Rozzano, Milan 20089, Italy; Department of Molecular and Translational Medicine, University of Brescia, Brescia 25123, Italy

**Keywords:** microRNA, Smooth muscle cell, Macrophages, Atherosclerosis, Gene expression/regulation, Vascular diseases

## Abstract

**Aims:**

MicroRNAs (miRNAs) regulate gene expression and are involved in various biological processes, including vascular homeostasis. Macrophages (Mϕs) and vascular smooth muscle cells (VSMCs) play key roles in vascular health and disease. However, the communication between Mϕs and VSMCs via miRNAs is not well understood. This study explores the transfer of miR-146a-5p from Mϕs to VSMCs and its role in atherosclerosis.

**Methods and results:**

Through unbiased miRNA-sequencing of cellular coculture, miR-146a-5p was identified as a potential messenger between Mϕs and VSMCs. This finding was validated using multiple experimental approaches, including the use of a fluorescent miR-146a-5p mimic and a sensor to document its transfer from Mϕs to VSMCs. Transfer occurred via gap junctions, especially when Mϕs were exposed to a pro-inflammatory stimulus. In VSMCs, miR-146a-5p promoted a contractile, proliferative phenotype and altered their metabolic and transcriptomic profiles, affecting genes involved in differentiation and cholesterol metabolism. Kruppel-like factor 4 (*Klf4*) was directly targeted by miR-146a-5p to modulate Serum Responsive Factor (SRF) activity and, hence, regulate genes such as Apolipoprotein E, 3-Hydroxy-3-Methylglutaryl-CoA Reductase, Thrombospondin 1, and Galectin 3. Of clinical importance, VSMCs from stenotic human plaque had an elevated miR-146a-5p level. A VSMC-specific sponge system targeting miR-146a-5p and, thus, hindering transfer from Mϕs, reduced plaque formation in a murine model of atherosclerosis.

**Conclusion:**

MiR-146a-5p is a key mediator of Mϕ-VSMC communication contributing to vascular disease, and is a potential therapeutic target for atherosclerosis.

**Time of primary review: 19 days**

**See the editorial comment for this article ‘Intercellular transport of miR-146a from macrophages to vascular smooth muscle cells: a novel mechanism and therapeutic target for atherosclerosis’, by P. Philpott *et al*., https://doi.org/10.1093/cvr/cvag102.**

## 1. Introduction

Atherosclerosis—a disease caused by lipid metabolism dysfunction triggering a maladaptive immune response and that impinges on the arterial wall and its components—is the primary cause of morbidity and mortality in the Western world.^1^ The disease usually progresses silently for decades, and by the time symptoms arise, significant damage has already occurred, inevitably leading to acute events.^2^ While recent advances in therapy have improved outcomes, its prevalence still remains high; moreover, with the continuous increase in aged individuals, the number of patients with the condition is rising.^3^ A new pharmacological approach to atherosclerosis, in particular through the modulation of the vascular inflammatory response and neointima formation, could significantly improve its prognosis.

Inflammation is a crucial factor in the initiation, progression, and outcome of atherosclerosis.^4^ Macrophages (Mϕs) are major inflammatory cells that convert into foam cells within the vessel walls where they are fundamental contributors to plaque formation and stability.^5^ Following endothelial injury, monocytes in peripheral blood infiltrate the vascular endothelium and differentiate into Mϕs, which subsequently become foam cells after taking up oxidized low-density lipoproteins and other lipids. By accumulating in atherosclerotic lesions, activated Mϕs contribute not only to the initiation of the pathology but also to its development and progression, ultimately leading to plaque instability and disruption.^6,7^

Activated Mϕs alter the biology of vascular cells, such as endothelial (ECs) and vascular smooth muscle (VSMCs) cells.^8^ Modulation of cell-to-cell communication within the vessel wall might reduce plaque formation^9,10^: Cellular cross-talk is primarily guided by cytokines, nitric oxide, and reactive oxygen species.^11,12^ Moreover, non-coding RNAs, such as microRNAs (miRNAs), were proved to play an essential role in the pathogenesis of atherosclerosis.^13,14^

One miRNA, miR-146, is highly expressed in Mϕs^15^ and mediates the inflammatory response in various inflammatory diseases.^16^ Different miR-146 family members, such as miR-146a and miR-146b, share similar functions in regulating the immune response. MiR-146a-5p is primarily involved in the regulation of inflammation and fine-tuning of the immune response.^17^ Its level is significantly increased in atherosclerotic plaque^18^ and in peripheral blood mononuclear cells from coronary artery disease patients.^19^ Moreover, its expression directly correlates with cardiometabolic risk factors,^20–22^ including levels of Trimethylamine-N-oxide (TMAO), a gut metabolite associated with increased risk of cardiovascular events.^23^ However, the mechanisms through which miR-146a-5p exerts its role in atherosclerosis is not yet fully understood. Here, we demonstrate that Mϕs communicate with other vascular cells, particularly VSMCs, via miR-146a-5p, modulating atherosclerosis development.

## 2. Methods

### 2.1 Animals

All animal procedures were performed according to institutional guidelines in compliance with national (4D.L. N.116, G.U., suppl. 40, 18-2-1992) and international law and policies (EEC Council-Directive 86/609, OJ L 358,1,12-12-1987; NIH Guide for the Care and Use of Laboratory Animals, US National Research Council-1996 and new directive 2010/63/EU) and approved by the Italian Ministry of Health (Authorization #816/2018-PR). ApoE^−/−^ mice (Charles River) were used for *in vivo* experiments. Special attention was paid to animal welfare and to minimize the number of animals used and their suffering. In case of discomfort animals were treated with Carprofen (5 mg/Kg/day) soministrated by intraperitoneal injection.

Disease model: Atherosclerosis was induced by feeding mice a hypercholesterolemic Western diet (WD) (Ca. #D12079B, Charles River) for 16 weeks (diet composition, g/Kg: 80 Mesh Casein, 195 g; DL-Methionine, 4 g; corn starch, 50 g; Maltodextrin 10, 100 g; sucrose, 341 g; Cellulose, 50 g; anhydrous milk fat, 200 g; corn oil, 10 g; mineral mix S10001, 35 g; Calcium Carbonate, 4 g; vitamin mix V10001, 10 g; Choline Bitartratem, 2 g; USPm Cholesterol, 1.5 g; and Ethoxyquin, 0.04 g).

After 16 weeks of WD, mice were euthanized by placing them in an isolated chamber filled with CO_2_ for 3–5 min (fill rate of 30–70% of the chamber volume per minute) and aortic roots fixed with 4% paraformaldehyde, OCT embedded, and sectioned for examination. Blood and a portion of aorta were collected for RNA extraction. Due to occasional tissue block, section damage, and folding, some mice had to be eliminated from some comparisons: in *Figure 7C* and Supplementary material online, *Figure S10C*, DSCR = 11, D146 = 11; in *Figure 7H*, DSCR = 11, D146 = 10; in Supplementary material online, *Figure S10D*, DSCR = 10, D146 = 8; in Supplementary material online, *Figure S10E*, DSCR = 11, D146 = 11; in Supplementary material online, *Figure S13C*, DSCR = 11, D146 = 11. Lesion evaluations were conducted on multiple aortic sections from the same mouse.

Infection of mice: A total of 1 × 10^7^ viral particles was systematically delivered with 3 intravenous tail injections every other day, as previously described.^24–26^ Groups were composed of equal numbers of female and male mice.

Blinding procedures: Mice were randomized with a specific ID number before procedures. At the end of the specific experiment, after sample processing and measurement evaluation (qPCR, immunohistochemistry, etc.), the correspondence between the ID number and genotype/treatment for each mouse was unveiled and data comparison then performed.

Power calculation: The number of mice per group was defined by performing a power analysis considering a type I error rate of 5% and a significant power of at least 80%.

### 2.2 Human samples

Human specimens were obtained from patients undergoing arterectomy of the internal carotid artery. Samples were recovered from the operating theatre and immediately processed by independent researchers. Specifically, the specimens were cleaned and separated into three parts: stenotic, medial and distal. Samples were fixed o/n in 4% paraformaldeide, and then moved to 100% ethanol prior to automatic processing for paraffin embedding. For subsequent analysis, measurements were obtained on multiple sections for each specimen, done in a blind fashion by two independent investigators, and the average values utilized for statistical analysis. For quantitation, images were acquired using Slide Scanner VS120 dotSlide and analyzed using Image J software.

The study was approved by Humanitas Research Hospital IRB (aut #3413) and adhered to the Declaration of Helsinki; informed consent was obtained from each patient. Patient characteristics are given in Supplementary material online, *Table S1*.

### 2.3 Coculture experiments

Polyethylene terephthalate (PET) membrane inserts with a 1.0 μm pore size (Ca. #MCRP12H48, Merck) were used to maintain cell types physically separate while allowing the exchange of small molecules, such as miRNAs. 150 000 Mϕs and 25 000 VSMCs at passage 5 were plated and maintained in complete Iscove's Modified Dulbecco’s Medium for 36 h in a humidified incubator at a controlled temperature of 37°C before collection.

For the validation of cell contact, Mϕs were stained with Calcein Red-Orange (Ca. #C34851, Life Technologies) and VSMCs with Blue Calcein (Ca. #C1429, Life Technologies); 36 h after coculture, a Z-stack acquisition was performed.

For fluorescence experiments, we first transfected Mϕs with 200 nM of fluorophore-conjugated (6-FAM) miR-146a-5p duplex RNA probe (custom designed, Ca. #VC30002N, Merck), using Lipofectamine RNAiMAX Reagent (Ca. #13778075, Life Technologies). The day after, we stained VSMCs with Calcein Red-Orange and added them to the transfected Mϕs in direct coculture, i.e. in physical contact. After overnight coculture, cells were washed with 1X PBS^−/−^ and fixed with 4% PFA for 15 min. Nuclei were stained with DAPI (Ca. #D1306, Life Technologies) and covers mounted with ProLong Diamond Antifade Mountant (Ca. #P36961, Life Technologies). Acquisition was performed with confocal microscopy (SP8, Leica).

### 2.4 Statistical analyses

Data are expressed as mean ± SD (standard deviation) calculated by Prism 7.0 software. Statistical calculations were performed on at least three independent experiments. Individual values were scanned for the presence of outliers using ROUT test. For the assumption of parametric tests, we evaluated the Gaussian distribution of values through Shapiro–Wilk normality test or Kolmogorov-Smirnov test. Statistical analyses were then performed with parametric unpaired Student’s *t*-test or Mann–Whitney test in the case of two groups of analysis (two-tailed). In the case of more than two groups of analysis, we used univariate ANOVA (1-way ANOVA corrected for multiple comparisons test) in the presence of one independent variable, or factorial design ANOVA (2-way ANOVA corrected for multiple comparisons test) to examine the effect of two factors on a dependent variable. Statistical significance was defined as *P* ≤ 0.05.

An extended method section is available in the Supplementary Material.

## 3. Results

### 3.1 Screening of miRNAs moving from Mϕs to VSMCs

**Figure 1:**
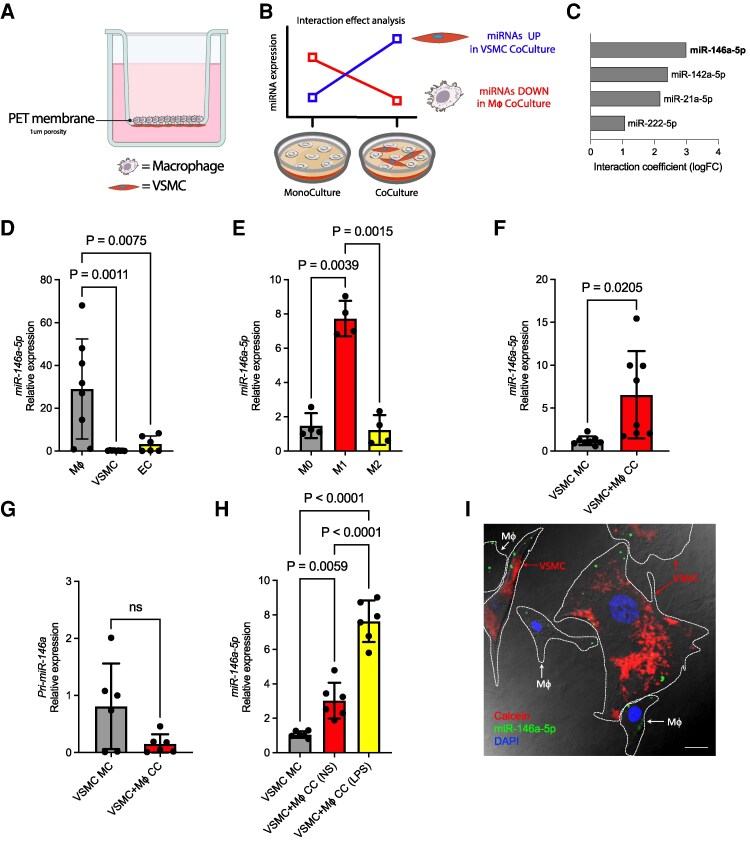
miR-146a-5p transfer from Mϕs to VSMCs. (*A*) Scheme of the coculture system for the study of cell-to-cell communication with cell types in direct contact. PET membrane inserts with a porosity of 1 μm were used, allowing the transfer of small molecules; Created with BioRender.com. (*B*) Schematic representation of the procedure for the identification of putative miRNAs transferred from Mϕs to VSMCs using miRNA-sequencing; Created with BioRender.com. (*C*) Interaction coefficient [adjusted *P*-value (FDR) < 10%] measured as logFC of putative transferred miRNAs. (*D*) Relative expression of mature miR-146a-5p measured by RT-qPCR in unstimulated Mϕs (*n* = 8), VSMCs (*n* = 9), and ECs (*n* = 6). (*E*) Relative expression of mature miR-146a-5p (*n* = 4) measured by RT-qPCR in unstimulated (M0), LPS-stimulated (100 ng/mL, M1), or IL-4-stimulated (20 ng/mL, M2) Mϕs for 48 h. (*F* and *G*) Relative expression of mature miR-146a-5p (F; *n* = 8) and pri-miR-146a (G; *n* = 6) measured in VSMCs after 36 h of coculture with Mϕs (CC) or in monoculture (MC). (*H*) Relative expression of mature miR-146a-5p (*n* = 6) measured in VSMCs after 36 h of coculture with either unstimulated (CC NS) or LPS-stimulated (CC LPS) Mϕs, or in monoculture (MC). (*I*) Representative confocal image of calcein-labelled VSMCs in coculture with Mϕs previously transduced with a fluorescent miR-146a-5p mimic. Merge of fluorescent images with bright-field image, with cell borders manually designed. Scale bar: 10 µm. For mature miRNAs evaluated via RT-qPCR, *U6 snRNA* or *mmu-SNORD65* were used as internal controls. For pri-miRNA evaluation via RT-qPCR, retro-transcribed as long RNA, *Ppia* was used as internal control. For ΔΔct analysis, we selected a single reference sample with a value of 1: Mϕ in 1D; M0 in 1E; VSMC MC in 1F, 1G, and 1H. Data represents mean ± SD. To compare means, ordinary one-way ANOVA with Tukey’s multiple comparisons test was used in A, B, and F, while unpaired *t*-test was used in D and E. ns, not statistically significant.

To study miRNA-mediated cell-to-cell communication between the two main cell types involved in atherosclerosis development, we established coculture (CC) systems using mouse primary Mϕs, derived from bone marrow, and mouse primary VSMCs, derived from aorta. For this purpose, we utilized PET membrane inserts with a 1 µm pore size. These inserts allow cell adhesion on both sides (*Figure 1A*), permit direct contact between the two plated cell populations (see Supplementary material online, *Figure S1A*), and allow the passage of small molecules, such as miRNAs.^27^ As a control, monoculture (MC) of each cell-type was performed under the same conditions as the CC. CCs were maintained for 36 h, after which cells were collected and miRNAs extracted. RNA quality was assessed, and then sequencing (seq) was performed using the SMARTer smRNA-seq KIT (Illumina) on the Next-Seq 500 platform.

For miRNA-seq analysis, we adopted an interactive approach to predict miRNAs transferred from Mϕs to VSMCs. We considered all possible cellular conditions: VSMCs and Mϕs in MC (VSMC MC and Mϕ MC), and VSMCs plus Mϕs in CC (VSMC + Mϕ CC). Differential expression analysis was performed to identify putative miRNAs transferred from Mϕs to VSMCs. To pinpoint these transferred miRNAs, we specifically evaluated the interaction effect between cell-type (VSMCs vs. Mϕs) and condition (CC vs. MC) in the context of differential expression. The analysis focused on miRNAs that were up-regulated in VSMCs in CC vs. MC conditions, while concurrently being down-regulated in Mϕs in CC vs. MC (*Figure 1B*). From this analysis, we identified four miRNAs [Adjusted *P*-value (FDR) < 10%] that putatively functioned as communication molecules between these two cell types. Among these, miR-146a-5p exhibited the strongest positive interaction coefficient, suggesting a role in the communication between Mϕs and VSMCs (*Figure 1C* and Supplementary File  *S1*).

Thus, we decided to elucidate the molecular mechanisms underlying the transfer of miR-146a-5p from Mϕs to VSMCs.

### 3.2 Validation of miR-146a-5p transfer from Mϕs to VSMCs

To confirm the preferential expression of miR-146a-5p in Mϕs compared with other cells of the inflamed vessel, we measured its basal expression in Mϕs, ECs, and VSMCs. As expected, miR-146a-5p expression was significantly higher in Mϕs compared with the other two cell types (*Figure 1D*). Next, we investigated whether miR-146a-5p expression varied among naïve (M0), pro-inflammatory [M1; Lipopolysaccharide-stimulated (LPS)], and anti-inflammatory (M2; IL4-stimulated) polarized Mϕs. There was a statistically significant increase in miR-146a-5p expression only in M1 Mϕs (*Figure 1E*), suggesting a role in multicellular inflammatory diseases, such as atherosclerosis.

To validate this finding and confirm the specificity of miR-146a-5p transfer from Mϕs to VSMCs rather than to other vascular cells, we performed similar CC experiments to that above, but with Mϕs and ECs. Reverse transcription quantitative polymerase chain reaction (RT-qPCR) was performed to measure the level of miR-146a-5p in the recipient cell (ECs or VSMCs) after CC with Mϕs. There was an increase in miR-146a-5p level in VSMCs (*Figure 1F*) but not in ECs (see Supplementary material online, *Figure S1B*), suggesting that this miRNA was specifically transferred to VSMCs.

To exclude *de novo* miRNA transcription induced by the proximity of the two cell types rather than to actual transfer of mature miRNA, we used RT-qPCR to measure the expression of the primary miRNA (pri-miR) in VSMCs. We found that increased expression of the mature miRNA in VSMCs was not accompanied by a corresponding increase in pri-miR expression, strongly indicating that the mature miRNA is directly transferred (*Figure 1G*). Furthermore, when VSMCs were in CC with M1 Mϕs, which stimulate miR-146a-5p expression in the donor cell (see Supplementary material online, *Figure S1C*, *D*), we observed a further increase in miR-146a-5p level in VSMCs (*Figure 1H*).

Finally, to further confirm that miR-146a-5p moves from Mϕs to VSMCs, we transduced primary Mϕs with a fluorescently labelled miR-146a-5p mimic and then cocultured them with VSMCs. Fluorescent signals indicative of the presence of the miRNA were detected in the receiving cells after CC (*Figure 1I* and Supplementary material online, *Figure S1E*), providing strong evidence that mature miR-146a-5p is transferred from Mϕs to VSMCs.

### 3.3 Gap junctions mediate the transfer of miR-146a-5p from Mϕs to VSMCs

**Figure 2:**
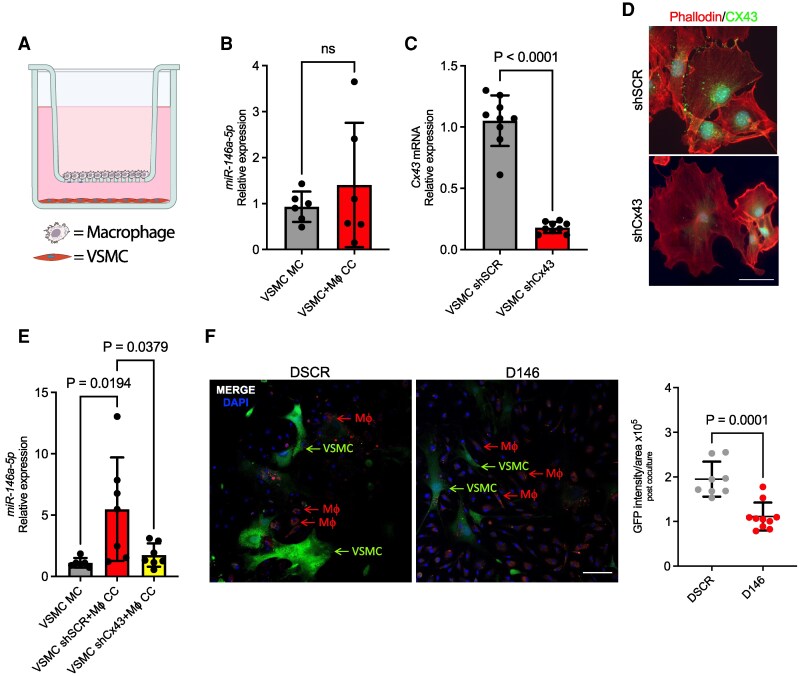
Transfer route of miR-146a-5p from Mϕs to VSMCs. (*A*) Scheme of the coculture system for studying indirect communication. In this case, cell types were plated separately: Mϕs in the PET insert, and VSMCs on the bottom of the plate; Created with BioRender.com. (*B*) Mature miR-146a-5p (*n* = 6) was measured in VSMCs after 36 h of indirect coculture with Mϕs or alone (CTR). (*C*) *Cx43* expression by RT-qPCR (*n* = 9) in VSMCs infected with lentiviral vectors targeting a scrambled (shSCR) or Connexin 43 (shCx43) sequence. (*D*) CX43 expression by fluorescent immunohistochemistry in VSMCs infected with lentiviral vectors targeting a scrambled (shSCR) or Connexin 43 (shCx43) sequence. (*E*) miR-146a-5p (*n* = 7) measured in VSMCs after 36 h of direct coculture with Mϕs (CC) or in monoculture (MC). In these experiments, we used VSMCs previously transduced with lentiviral vectors harbouring Connexin43 shRNA (shCx43) or a scrambled sequence (shSCR). (*F*) Representative images of VSMCs transduced with a vector expressing the GFP gene controlled by the SM22 promoter and with a synthetic 3′UTR carrying scrambled (DSCR) or decoy (D146) sequences of miR-146a-5p cocultured with Calcein-labelled Mϕs (*n* = 3; at least 30 cells were quantified in each experiment). Scale bar: 100 µm. For mature miRNA evaluated via RT-qPCR, *U6 snRNA* or *mmu-SNORD65* were used as internal controls. For *Cx43* evaluation via RT-qPCR, *Ppia* was used as internal control. For ΔΔct analysis, we selected a single reference sample with a value of 1: VSMC MC in 2B and 2D; VSMC shSCR in 2C. Data represents the mean ± SD. To compare means, unpaired *t*-test was used in B, C, and E, while ordinary one-way ANOVA with Tukey's multiple comparisons test was used in D. ns, not statistically significant.

To investigate the molecular mechanism by which miR-146a-5p moves from Mϕs to VSMCs, we evaluated whether this transfer occurred via direct cell contact or an indirect interaction (i.e. secretion). First, we set up CCs in which donor and recipient cells were not in contact: to this end, donor cells were plated on the top surface of the PET insert whereas recipient cells were plated on the bottom of the wells to evaluate potential paracrine transfer (*Figure 2A*). As before, indirect CCs were maintained for 36 h before measuring the miR-146a-5p level in VSMCs. In this setup, we did not observe any significant difference in levels between control VSMCs (VSMC MC) and those indirectly cocultured with Mϕs (*Figure 2B*). This result is clearly indicative that miR-146a-5p is transferred only through direct contact between the two cell types.

Having established that direct contact is necessary for miR-146a-5p transfer, we investigated the molecular mechanism by which it occurs. Among direct mechanisms of miRNA transfer, gap junctions are one plausible route.^28^ To test this hypothesis, we generated lentiviral vectors (shCx43) to specifically silence Connexin 43 (CX43), a key protein involved in gap junction formation.^29^ Since primary Mϕs are terminally differentiated, are resistant to viral infection, and do not replicate in culture, and since gap junctions require CXs from both communicating cells, we silenced *Cx43* only in VSMCs, which is sufficient to prevent gap junction formation. *Cx43* silencing in VSMCs was confirmed by RT-qPCR, with 80% reduction in expression at RNA and protein levels (*Figure 2C, D*); moreover, *Cx43*-silenced VSMCs did not show any modulation in terms of shape or size (*Figure 2D* and Supplementary material online, *Figure S1F*). The gap junction-deficient VSMCs were then placed with Mϕs in direct contact CC for 36 h. VSMCs transduced with a scrambled sequence (shSCR) were used for control. No miR-146a-5p transfer was observed in experiments using shCx43-VSMCs (*Figure 2E*), confirming the hypothesis that the transfer of miR-146 occurs only in the presence of functional gap junctions.

Next, we evaluated whether the transferred miRNA had functional consequences. To this end, we engineered a vector in which three complementary sequences for miR-146-5p were cloned as a 3′ untranslated region (UTR) downstream of the Green Fluorescence Protein (GFP) gene, with GFP gene expression controlled by the VSMC-specific promoter Transgelin (SM22)^30^ (see Supplementary material online, *Figure S2A*). The efficacy of this construct was tested by co-transfecting HEK293T cells with: (ⅰ) a vector carrying a decoy sequence for miR-146a-5p (D146) or a scrambled sequence (DSCR) after the GFP (a decoy for miR-143-3p (D143) was used as a further control); and (ⅱ) a plasmid expressing either miR-146a-5p or a scrambled sequence (SCR) (see Supplementary material online, *Figure S2B*).^25^ Fluorescence microscopy confirmed that the presence of miR-146a-5p reduced the GFP signal only in the D146 system (see Supplementary material online, *Figure S3A*).

Then, we utilized the D146 construct as a sensor to demonstrate the functional transfer of endogenous miR-146a-5p from Mϕs to VSMCs. To this end, we transduced naïve VSMCs with a lentiviral vector expressing the D146 system or the relative control (DSCR). No difference in the GFP signal was measured in VSMCs prior to CC (see Supplementary material online, *Figure S3B*). However, when these cells were cocultured with primary Mϕs labelled with Calcein, a statistically significant reduction in GFP intensity was detected in VSMCs carrying the D146 sequence. This finding confirms the functional transfer of endogenous miR-146a-5p from Mϕs to VSMCs (*Figure 2F* and Supplementary material online, *Figure S3C*).

In conclusion, miR-146a-5p is directly transferred from Mϕs to VSMCs via gap junctions.

### 3.4 Modulation of VSMC features induced by miR-146a-5p

**Figure 3:**
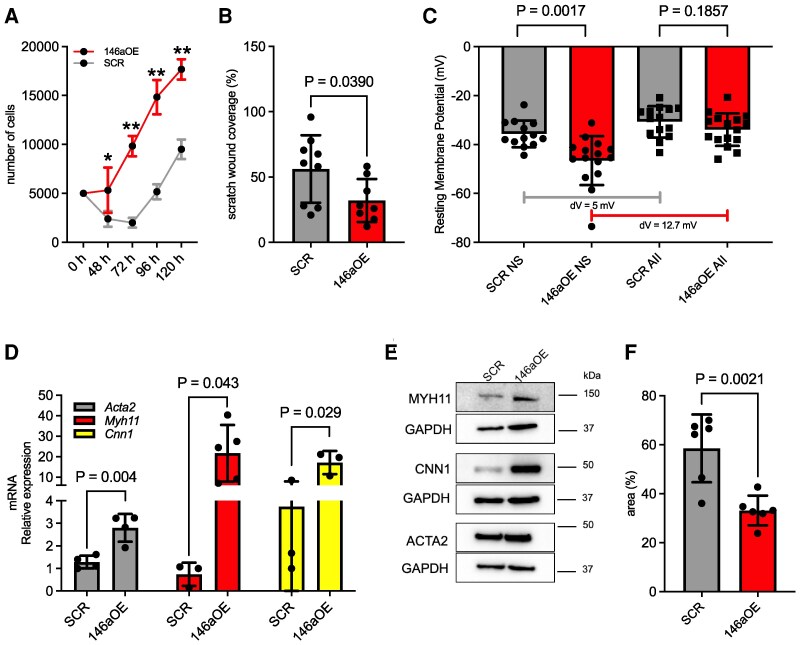
Phenotypic analysis of miR-146a-5p-overexpressing VSMCs. (*A*) Growth curve of VSMCs overexpressing miR-146a-5p (146aOE) vs. cells expressing a scrambled miRNA (SCR) (*n* = 3). (*B*) Migration assay on 146OE VSMCs vs. SCR cells (*n* ≥ 8). (*C*) Resting membrane potential data (*n* ≥ 13) recorded from control SCR and 146aOE cells, in the basal condition (NS) or following stimulation with Angiotensin II (AII). (*D*) Expression of contraction markers in SCR and 146aOE cells, measured by RT-qPCR (*n* ≥ 3). (*E*) Representative Western blots showing differentiation markers (ACTA2, actin alpha 2; MYH11, myosin heavy chain 11; and CNN1, calponin 1) in SCR and 146aOE cells. (*F*) Quantification of collagen contraction after 24 h in SCR and 146aOE cells (*n* = 6). For evaluation of contraction markers via RT-qPCR, *Ppia* was used as internal control. For ΔΔct analysis, we selected a single reference sample of SCR with a value of 1. Data represents the mean ± SD. To compare means, unpaired Student's *t*-test was used in *B*, *C*, *D*, and *F*, while 2-way ANOVA with Sidak's multiple comparisons test was used in *A*. * *P* = 0.002, ** *P* < 0.0001; dV, voltage difference.

The role of miR-146a-5p as an immune regulator has been well characterized.^15^ However, the impact of the transfer from Mϕs to VSMCs is not known. To address this question and elucidate the molecular functions of miR-146a-5p in VSMCs, we utilized a previously validated gain-of-function approach using a lentiviral vector for the expression of mature miR-146a-5p (see Supplementary material online, *Figure S2B*).^25^ We first tested the efficacy of the method by measuring miR-146a-5p overexpression (146aOE) in transduced VSMCs (see Supplementary material online, *Figure S3D*). Subsequently, we performed various cellular assays to investigate the effects of miR-146a-5p on VSMCs. 146aOE VSMCs had a higher proliferation rate than did SCR VSMCs; there were no differences in term of cell viability, as shown by the growth curve of trypan blue-labelled cells (*Figure 3A*).

To evaluate whether this observation held true also in physiological conditions, i.e. in non-OE cells, we plotted the proliferation curve of primary naïve VSMCs after CC with Mϕs. These cells had a similar trend of increased proliferation, a finding validating the 146aOE data (see Supplementary material online, *Figure S4A*). Additionally, when VSMCs were cocultured with Mϕs pre-treated with LPS, there was a higher level of miR-146a-5p transfer (*Figure 1H*) and, again, a correlated increased proliferation rate (see Supplementary material online, *Figure S4B*). Of note, 146aOE VSMCs exhibited a reduced migratory capacity, as measured by a scratch assay (*Figure 3B* and Supplementary material online, *Figure S4C*). This reduction in migration suggested that VSMCs with a higher level of miR-146a-5p might be more differentiated.

To test whether miR-146a-5p altered the electrophysiological and contractile properties of VSMCs, we conducted patch-clamp experiments.^25^ 146aOE VSMCs had a significantly more hyperpolarized membrane potential under the basal condition than did control cells (*Figure 3C*). Statistical difference disappeared when the cells were exposed to the contractile stimulus Angiotensin II (AII). However, AII-treated 146aOE VSMCs exhibited a greater range of depolarization compared with SCR VSMCs. Specifically, a membrane potential of −35.7 mV was recorded in SCR VSMCs under the non-stimulated condition, and this shifted to −30.7 mV in the presence of AII (dV = 5 mV). In 146aOE VSMCs, the membrane potential shifted from −46.6 mV in the non-stimulated condition to −33.9 mV in the presence of AII (dV = 12.7 mV). No differences in membrane capacitance were observed (see Supplementary material online, *Figure S4D*), and, as expected, cell size remained unchanged across different groups (see Supplementary material online, *Figure S4E*).

In conclusion, this set of electrophysiology experiments confirmed that miR-146a-5p induced electrical remodelling in VSMCs, manifested as greater depolarization in the presence of AII, which could result in a stronger contraction force in 146aOE cells. Consistent with these results, we observed increased expression of VSMC contraction markers at the RNA (*Figure 3D*) and protein (*Figure 3E* and Supplementary material online, *Figure S4F*, *H*) levels. Similar findings, in terms of increased expression of differentiation markers, and specifically of Smooth Muscle Actin (*Acta2*) and Myosin Heavy Chain 11 (*Myh11*), were also observed in primary naïve VSMCs cocultured with Mϕs (see Supplementary material online, *Figure S4I*), further supporting the resemblance between 146aOE cells and VSMCs cocultured with Mϕs. Finally, these findings were also corroborated by a plug collagen assay, which demonstrated enhanced contractile capacity in 146aOE VSMCs (*Figure 3F*).

This series of experiments clearly demonstrates that miR-146a-5p triggers a contractile, yet proliferative, phenotype in VSMCs.

### 3.5 Gene expression analysis of VSMCs expressing miR-146a-5p

To elucidate the specific molecular mechanisms involving miR-146a-5p in VSMCs, we performed bulk RNA sequencing on three samples of 146aOE VSMCs and control SCR cells. Principal component analysis (PCA) of transcript counts revealed that these two groups clearly segregated on the first component (see Supplementary material online, *Figure S5A* and Supplementary File  *S2*), which explains 89.73% of the variance, suggesting that miR-146a-5p induces a reprogramming of the VSMC transcriptome.

**Figure 4:**
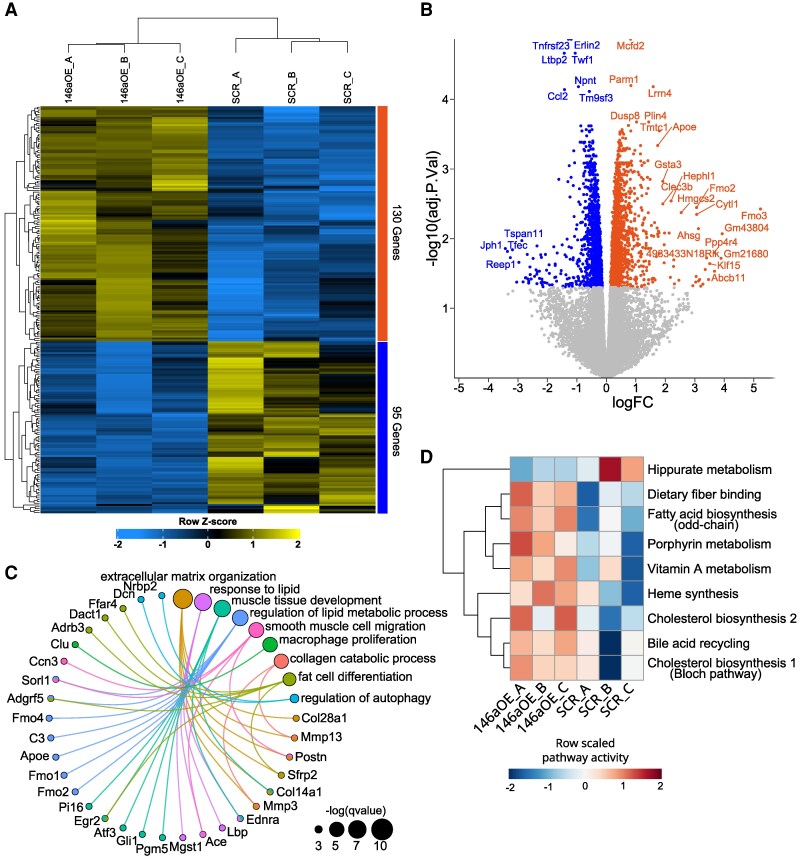
Transcriptional profile of miR-146-overexpressing VSMCs. (*A*) Heatmap of differentially expressed genes with logFC > |1| and adj.P.Val <0.05 in VSMCs overexpressing miR-146a; the colour bar reflects the scaled expression values, and the dendrograms represent the Euclidean distances among samples and genes. (*B*) Volcano plot of differentially expressed genes in miR-146aOE VSMCs; up-regulated genes are in orange, and down-regulated ones are in blue (adj.P-value < 0.05); the *x*-axis shows the logFC values computed by the limma package, and the *y*-axis shows the -log10 (adj.P.Val). (*C*) Circos plot of the core enrichment genes with logFC > 1 and adj.P.Val < 0.05, along with the enriched pathway terms. (*D*) Heatmap of the reconstructed metabolic pathways from METAFlux algorithm of VSMCs overexpressing miR-146a (146aOE) or scrambled sequences (SRC); the colour bar reflects the pathway activity score calculated as mean of fluxes of associated reactions. Only significantly different pathways (*P* < 0.05) for the Kruskal-Wallis test between the two groups are shown.

Differential gene expression analysis identified a total of 1122 genes with altered expression, including 130 down-regulated and 95 up-regulated genes. These results are depicted in *Figure 4A* and *B*, which respectively show a hierarchically clustered heatmap illustrating the clear separation of biological replicates based on significantly perturbed genes, and a volcano plot highlighting the significant differentially expressed genes, with up-regulated genes coloured in orange and down-regulated genes in blue. These findings demonstrate the transcriptional reprogramming triggered by miR-146a-5p overexpression in VSMCs.

Gene Set Enrichment Analysis (GSEA) of the perturbed genes identified in the GO biological process database 231 pathways that were differentially enriched between the two groups (*q*-value < 0.05). Among the top enriched pathways in the 146aOE group, we selected nine closely related pathways that reflected the changes in cellular plasticity of 146aOE VSMCs: extracellular matrix organization (GO:0030198), response to lipid (GO:0030198), muscle tissue development (GO:0060537), regulation of lipid metabolic process (GO:0019216), smooth muscle cell migration (GO:0014909), collagen catabolic process (GO:0030574), macrophage proliferation (GO:0061517), fat cell differentiation (GO:0045444), and regulation of autophagy (GO:0010506). These biological pathways are presented in *Figure 4C* as a circos plot, in which enriched pathways are represented by circles with sizes reflecting the significance of the pathways and linked to the core enrichment genes significantly up-regulated in 146aOE cells. This analysis confirmed, at the transcriptomic level, several biological features of 146aOE VSMCs, including modulation of migration and proliferation (GO:0014909 and GO:0061517 respectively). Supplementary material online, *Figure S5B* shows the running score plot of each pathway, highlighting the significance of the enrichments.

Among the modulated GO biological processes shown in *Figure 4C*, several were associated with lipid metabolism and adipose cells (GO:0030198, GO:0019216, and GO:0045444). This observation prompted us to investigate whether miR-146a-5p induced a metabolic switch in VSMCs. To address this, we analyzed the entire transcriptional profile of each sequenced sample (146aOE, *n* = 3; SCR, *n* = 3). The METAFlux algorithm^31^ was applied to identify genes involved in metabolic reactions, integrating them into a comprehensive metabolic profile to predict putative derived metabolites, including intra- and extra-cellular secreted molecules. This bioinformatics workflow integrated all data to define the activity of specific metabolic pathways for each sample. Using this approach, we identified all statistically modulated metabolic pathways in 146aOE VSMCs vs. control SCR samples. The heatmap in *Figure 4D* clearly illustrates the separation between the two groups based on their putative metabolic activity. Notably, there was a statistically significant increase in cholesterol biosynthesis pathways.

Taken together, these findings highlight a substantial impact of miR-146a-5p on the regulation of VSMC metabolism.

### 3.6 Metabolic and functional effects of miR-146a-5p in VSMCs

**Figure 5:**
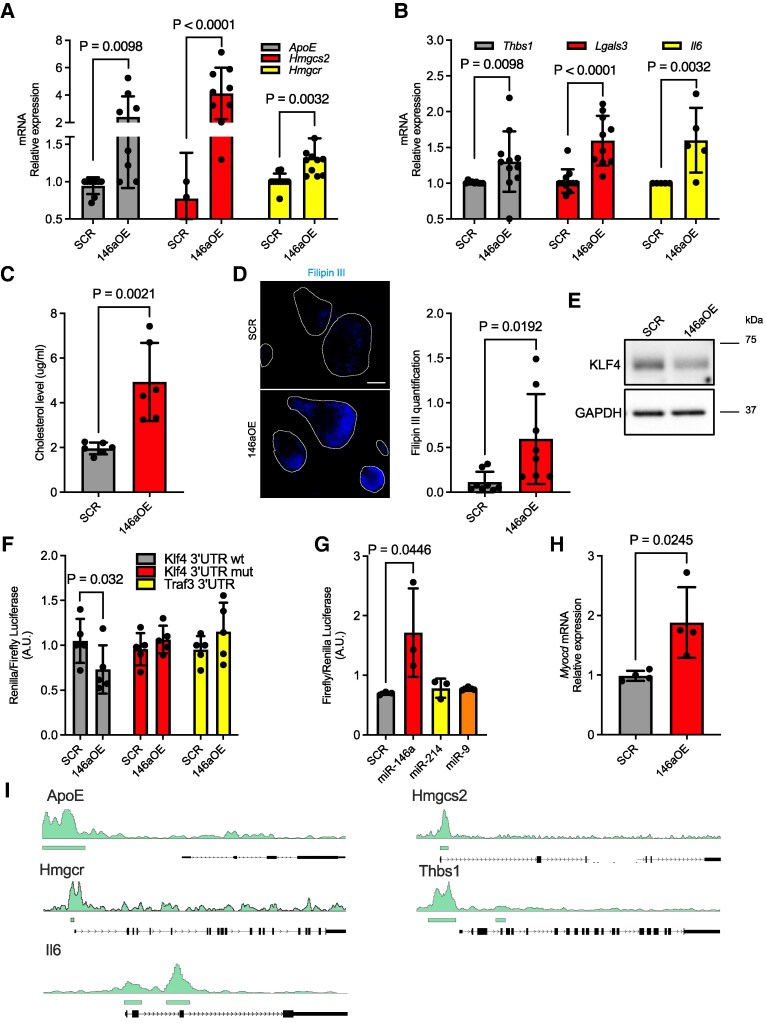
Cholesterol synthesis modulation and gene target identification in miR-146-overexpressing VSMCs. (*A* and *B*) Gene expression analysis for genes correlated with cholesterol biosynthesis (*A*; *n* ≥ 8) and with Mϕ phenotype (*B*; *n* ≥ 8), comparing control (SCR) vs. 146aOE VSMCs. (*C*) Quantification of total cholesterol produced by control (SCR) and 146aOE VSMCs measured by mass spectrometry (*n* = 6). (*D*) Representative image of Filipin III-labelled SCR and 146aOE VSMCs for cholesterol and relative quantification (*n* = 8, for each experiment 30 cells were quantified). Scale bar: 10 µm. (*E*) Representative Western blot showing KLF4 expression in SCR and 146aOE cells. (*F*) Target luciferase reporter assays using vectors encoding putative target sites in the 3′UTR (Klf4 wt, Klf4 mut, and Traf 3). VSMCs were transiently co-transfected with either miR-146a-5p (146aOE) or scrambled control (SCR), and with wild-type 3′UTR reporter plasmids for *Klf4* or *Traf3*. Renilla luciferase activity was measured 48 h after transfection. The results are normalized to firefly luciferase values (*n* = 5). (*G*) SRF luciferase reporter assays: VSMCs were transiently co-transfected with mimics for miR-146a-5p, miR-214-3p, miR-9-5p, or a scrambled (SCR) sequence, and Acta2 reporter plasmids. Luciferase activity was measured 72 h after transfection. The results are normalized to renilla values (*n* = 3). (*H*) RT-qPCR analysis for the expression of *Myocd* in SCR and 146aOE VSMCs (*n* = 4). (*I*) Enriched SRF ChIP-seq peaks of VSMCs in Mϕs/lipidic marker genes modulated by miR-146a-5p. Horizontal green bars indicate the statistical enriched SRF binding sites. For gene expression evaluation via RT-qPCR, *Ppia* was used as internal control. For ΔΔct analysis, we selected a single reference sample of SCR with a value of 1 for 5*A*, 5*B*, and 5*H*. Data represents the mean ± SD. To compare means, unpaired Student's *t-*test was used in *A*, *B*, *C*, *D*, *F*, and *H*; 1-way ANOVA with Tukey's multiple comparisons test was used in *G*.

VSMCs expressing miR-146a-5p exhibited a distinct phenotype characterized by increased contractility and proliferation. However, RNA-seq data suggested a transition toward a modulated metabolic state, which was further, validated using Metaflux analysis. To investigate whether this modulated metabolic state resembled that of Mϕs, we measured the expression levels of genes classically associated with cholesterol metabolism and Mϕ transition. RT-qPCR analysis revealed significant up-regulation of genes involved in cholesterol metabolism and transport, including Apolipoprotein E (*ApoE*), a key player in cholesterol transport^32^; 3-Hydroxy-3-Methylglutaryl-CoA Synthase 2 (*Hmgcs2*), known for its role in ketogenesis^33^ as well as in the initial synthesis step of Acetoacetyl-CoA, a precursor of cholesterol^34^; and 3-Hydroxy-3-Methylglutaryl-CoA Reductase (*Hmgcr*), a critical enzyme in cholesterol biosynthesis and the target of statins^35^ (*Figure 5A*). For Mϕ transition, we analyzed the expression of Thrombospondin 1 (*Thbs1*), whose expression correlates with a pro-inflammatory phenotype^36^; Galectin 3 (*Lgals3*), a marker of Mϕ activation^37^; and Interleukin 6 (*Il6*), a well-known pro-inflammatory cytokine^38^ (*Figure 5B*). Next, we evaluated the overall cholesterol level in 146aOE VSMCs by mass spectrometry, observing a marked presence of cholesterol in these cells (*Figure 5C*). This finding was corroborated by direct fluorescent staining of cholesterol with Filipin III (*Figure 5D*, Supplementary material online, *Figure S6A*, *B*).

Transcriptomic data also suggested that miR-146a-5p might influence autophagy (*Figure 4C*), a critical process in atherosclerosis development.^39^ To investigate this, we assessed whether autophagy was altered in 146aOE VSMCs. RT-qPCR analysis revealed no significant modulatory changes in the expression of the autophagy genes Autophagy Related 3 (*Atg3*), Beclin 1 (*Becn1*), and Microtubule Associated Protein 1 Light Chain 3 Alpha (*Lc3a*) when miR-146a-5p was overexpressed in VSMCs (see Supplementary material online, *Figure S7A*). These findings were further supported by a luminescence assay in which SCR and 146aOE VSMCs were transduced with an LC3 reporter vector. No difference was observed in the luminescent signal between the two conditions (see Supplementary material online, *Figure S7B*).

In conclusion, VSMCs overexpressing miR-146a-5p are contractile cells with enhanced cholesterol production, elevated expression of Mϕ-associated genes, and a pro-atherosclerotic phenotype.^40^

### 3.7 Identification of miR-146a-5p targets in VSMCs

Having established that the transfer of miR-146a-5p from Mϕs exerts significant biological effects on VSMCs, modulating phenotypic and metabolomic characteristics, we sought to identify the targets of this miRNA in VSMCs. Using the bioinformatics tool TargetScan (https://www.targetscan.org/), we searched for potential target genes. Among these, we selected Krüppel-Like Factor 4 (*Klf4*) as a potential binding partner of miR-146a-5p (see Supplementary material online, *Figure S8A*) for further investigation, given its fundamental role in VSMC biology.^41^ Transduction of primary VSMCs with a lentiviral vector encoding miR-146a-5p reduced the abundance of the target protein (*Figure 5E* and Supplementary material online, *Figure S8B*), although no difference was observed at the mRNA level (see Supplementary material online, *Figure S8C*), as often observed in miRNA activity.^42^ A luciferase assay with 3′ UTR binding sites confirmed that *Klf4* is directly targeted by miR-146a-5p, while two controls—the mutated seed sequence and a not-predicted target 3′ UTR (namely, TNF Receptor Associated Factor 3)—were not (*Figure 5F*).

KLF4 regulates several VSMC genes through different mechanisms. Notably, it negatively modulates the activity of Serum Responsive Factor (SRF) by competing with the SRF cofactor Myocardin (MYOCD).^43,44^ This mechanism is fundamental in VSMC biology.^41^ We therefore investigated whether miR-146a-5p expression in VSMCs affected SRF activity. To do this, we co-transfected VSMCs with a plasmid expressing miR-146a-5p and a luciferase reporter vector for SRF activity.^45^ 72 h post-transfection, cells were collected and luciferase activity measured. We observed, as expected, an increase in SRF activity due to miR-146a-5p-mediated inhibition of KLF4 expression, while control miRNAs had no effect (*Figure 5G*). Additionally, 146aOE VSMCs exhibited a statistically significant increase in expression of *Myocd* (*Figure 5H*).

To corroborate these findings and connect them to the observed phenotype of 146aOE VSMCs, we analyzed chromatin immunoprecipitation sequencing data for SRF in VSMCs (GSE112417).^46,47^ SRF binding peaks were observed, as expected, in genes associated with VSMC differentiation [e.g. *Acta2*, Calponin (*Cnn1*), *Myh11*, and Transgelin (*Tagln*)] (see Supplementary material online, *Figure S8D* and Supplementary File  *S3*). However, we also found significant SRF enrichment in genes up-regulated in 146aOE VSMCs and involved in cholesterol metabolism (*ApoE*, *Hmgcs2*, and *Hmgcr*) or associated with a pro-inflammatory Mϕ phenotype (*Thbs1* and *Il6*) (*Figure 5I* and Supplementary File  *S3*). These data provide molecular evidence supporting a distinct VSMC phenotype induced by the miRNA.

Thus, *Klf4* is a key VSMC phenotype-modulating target of miR-146a-5p.

### 3.8 Spatial localization of miR-146a-5p in human atherosclerotic plaque

Atherosclerotic plaque is a highly complex structure; it is classified as advanced by histological criteria when lipid accumulation and matrix components, including minerals, are associated with structural disorganization and deformity of the arterial wall.^48^ Histological sections of atherosclerotic plaque are often discontinuous due to the complex and irregular nature of the tissue and specific cellular distribution. Therefore, understanding the spatial localization and distribution of miR-146a-5p in different areas of pathological human vessel is of particular interest.

**Figure 6:**
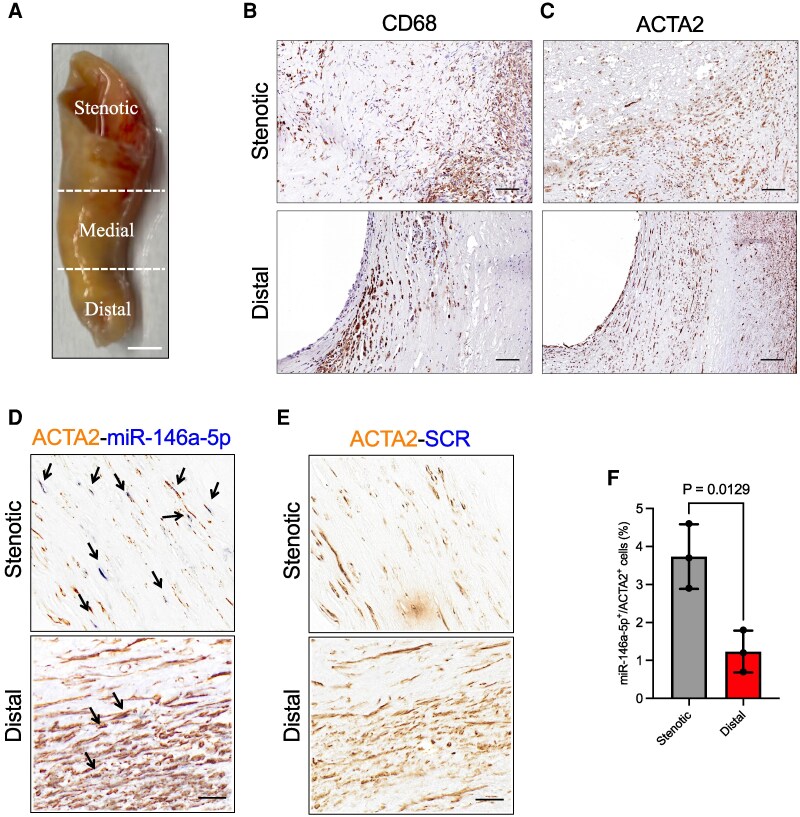
Spatial localization of miR-146a-5p in human plaque. (*A*) Representative image of an explanted atherosclerotic plaque from human carotid artery, divided in three parts, going from the least to the most stenotic. Scale bar: 0.4 cm. (*B* and *C*) Representative images of CD68 and ACTA2 staining of the distal and stenotic sections of the plaque. Scale bar: 100 μm. (*D* and *E*) Representative images of multiplex *in situ* staining using a specific probe for miR-146a-5p and a scrambled (SCR) one as control, and an antibody against ACTA2. Scale bar: 100 μm. (*F*) Quantification of the percentage (%) of miR-146a-5p^+^ VSMCs. The data were derived from three plaques. Data represent mean ± SD. To compare means, unpaired Student's *t-*test was used in *F*.

To perform this task, we divided human plaques into three parts: distal, medial, and stenotic (*Figure 6A*). We then performed hematoxylin-eosin staining on the distal and stenotic sections to assess pathological tissue alteration and cellularity (see Supplementary material online, *Figure S9*). Next, we stained for Scavenger Receptor Class D, Member 1 (CD68) to visualize Mϕs in the tissue (*Figure 6B*), and for ACTA2 to visualize VSMCs (*Figure 6C*). We then performed multiplex colorimetric staining combining *in situ* hybridization for miR-146a-5p with immunostaining for ACTA2 (*Figure 6D*); a scrambled (SCR) probe was used as a negative control (*Figure 6E*). The analysis revealed that both parts of the analyzed plaques (stenotic and distal) presented, with differences in size, classical atherosclerotic areas and zones conserving a pseudo-physiological medial section (*Figure 6C*). However, the percentage of miR-146a-5p^+^ VSMCs was markedly increased in pathological areas of the stenotic region compared the distal ones (*Figure 6F*).

These data indicate the presence of increased miR-146a-5p, potentially from transfer from Mϕs, in VSMCs in human plaque.

### 3.9 Inhibition of miR-146a-5p-induced VSMC effects in ApoE^−/−^ mice

To investigate whether modulating the activity of Mϕ-derived miR-146a-5p in VSMCs impacts atherosclerosis development, we utilized the miR-146a-5p decoy lentiviral vector described earlier (see Supplementary material online, *Figure S2B*). The sponge expression system is regulated by the SM22 promoter,^30^ ensuring its activity is restricted only to the VSMC layer. First, the efficacy and specificity of this system were confirmed in VSMCs *in vitro*: indeed, delivery induced a strong GFP signal exclusively in VSMCs but not in other vascular cells, such as ECs and Mϕs (see Supplementary material online, *Figure S10A*).

**Figure 7:**
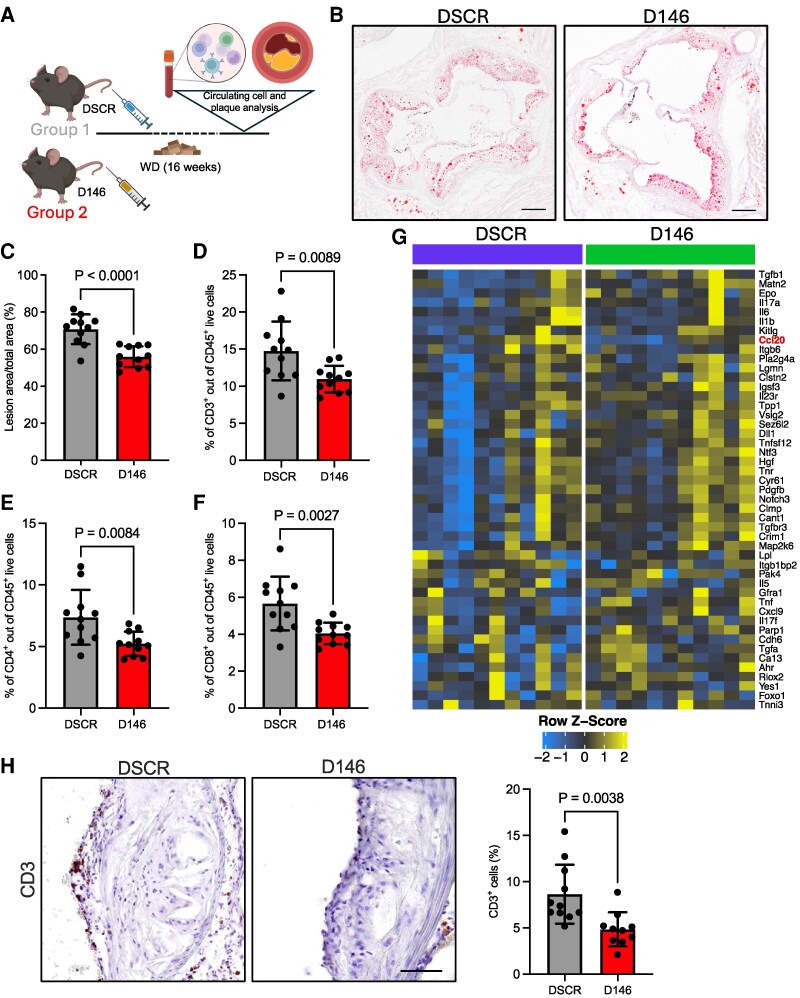
*In vivo* effects of specific miR-146a-5p inhibition in VSMCs during atherosclerosis development. (*A*) Schematic representation of the experiment performed in ApoE^−/−^ mice. Animals were infused with a lentiviral vector carrying D146 or DSCR as control and then fed a Western diet (WD) for 16 weeks; Created with BioRender.com. (*B*) Representative red-oil staining images from DSCR and D146 animals. Scale bar: 200 µm. (*C*) Quantification of lesion considering the ratio lesion area/total plaque areas (DSCR, *n* = 11; D146, *n* = 11). (*D*–*F*) Quantification of circulating CD3^+^, CD4^+^, and CD8^+^ cells in D146 and DSCR treated animals, measured by FACS analysis (*n* = 11). (*G*) Analysis of circulating factors in serum of treated animals, using the Olink technology exploratory panel (*n* = 9). (*H*) Representative CD3 staining images of DSCR and D146 animals and relative quantification of the percentage of CD3^+^ cells (DSCR, *n* = 11; D146, *n* = 10). Scale bar: 50 µm. Data represent mean ± SD. To compare means, unpaired Student’s *t-*test was used.

Next, we injected this vector (D146) or the control (DSCR) in ApoE^−/−^ mice; the mice were then fed a WD for 16 weeks, after which plaque formation was evaluated in aortic root cross-sections (*Figure 7A*). Reduced expression of miR-146a-5p in the VSMC layer of aortas, which had their adventitia and intima layers physically removed, was confirmed by RT-qPCR (see Supplementary material online, *Figure S10B*). Notably, D146-treated mice exhibited a significant reduction in plaque size, as measured by ratio lesion area/total area and absolute total plaque area (*Figure 7B, C* and Supplementary material online, *Figure S10C*). There were no differences in the percentage of ACTA2 positivity (see Supplementary material online, *Figure S10D*) or fibrotic area (see Supplementary material online, *Figure S10E*, left graph), but there was a reduction in necrotic plaque area (see Supplementary material online, *Figure S10E*, right graph). We also measured the circulating levels of total, LDL, and HDL cholesterol: while there were no differences in total and LDL cholesterol, a statistically significant increase of HDL level was observed, a finding consistent with a general protective effect of D146 treatment (see Supplementary material online, *Figure S10F*, *H*).

Given the observed reduction in plaque formation, we investigated whether modulating the transfer of miR-146a-5p from Mϕs to VSMCs *in vivo* might also influence circulating immune cells. To evaluate this, we measured the percentage of specific CD45^+^ cell subtypes. D146 mice had a significant reduction in CD3^+^ cells (*Figure 7D*), including lower levels of conventional and regulatory (Treg) subsets of CD4^+^ T cells (*Figure 7E*; Supplementary material online, *Figure S11A*, *B*), CD8^+^ T cells (*Figure 7F*), and Natural Killer (NK) cells (see Supplementary material online, *Figure S11C*). No significant differences were observed in other cell subsets (see Supplementary material online, *Figure S11D*, *F*).

To further understand the reduction in circulating immune cells in D146-treated mice and to link this effect to the reduced miR-146a-5p level in VSMCs, we analyzed several secreted factors in serum, using Olink proximity extension technology. We run exploratory (94 analyzed elements) and cytokine-specific (48 analyzed elements) panels. D146 mice had reduced CCL20 [exploratory panel: DSCR, 10.42 ± 0.94 npx (normalized protein expression); D146, 9.6 ± 0.78 npx; *P* = 0.046] (*Figure 7G*, Supplementary material online, *Figure S12A* and Supplementary File  *S4*) and CCL22 (cytokine-specific panel: DSCR, 446 ± 136 pg/mL; D146, 336 ± 70 pg/mL; *P* = 0.048) (see Supplementary material online, *Figure S12B* and Supplementary File  *S5*) vs. DSCR controls.

To identify whether the modulation of these cytokines directly depended on VSMCs, we measured their expression in 146aOE VSMCs by RT-qPCR. There was a marked increase of *Ccl20* expression in 146aOE VSMCs, while no variation was detected for *Ccl22* (see Supplementary material online, *Figure S12C*). These data support the hypothesis that the down-regulation of *Ccl20* in the serum of D146 mice is directly dependent on the modulation of miR-146a-5p in VSMCs, while the modulation of *Ccl22* is an indirect effect.

CCL20—a cytokine known to be produced by VSMCs^49,50^ plays a critical role in monocyte and leukocyte trafficking to inflamed sites by attracting these cells via their CCR type 6 (CCR6) receptor.^51,52^ This could explain, for instance, the reduced level of circulating T cells in D146 mice, contributing as part of the complex effect in global plaque formation.

Consistent with this, we also observed a decreased percentage of CD3^+^ T cells (*Figure 7H* and Supplementary material online, *Figure S13A*) and F4/80^+^ Mϕs (see Supplementary material online, *Figure S13B*, *C*) in the plaques of D146 mice vs. DSCR mice.

These results indicate that *in vivo* inhibition of Mϕ-derived miR-146-5p activity directly in VSMCs reduces atherosclerotic plaque formation, a desirable outcome for preventing plaque rupture and subsequent cardiovascular events.

## 4. Discussion

In this study, we identify miR-146a-5p as a novel mediator of intercellular communication between Mϕs and VSMCs, with significant implications for the pathogenesis of atherosclerosis. This discovery adds a new layer to our understanding of vascular inflammation and remodelling, highlighting miR-146a-5p as a potential therapeutic target.

MiR-146a-5p has been extensively studied in the context of immune regulation, particularly in Mϕs, where it serves as a negative feedback regulator of NF-κB signalling and inflammatory cytokine production.^15^ Its up-regulation in atherosclerotic lesions^18,53,54^ and correlation with cardiovascular risk factors such as TMAO levels and systemic inflammation have been well documented.^23^ However, its role in non-immune vascular cells has remained largely unexplored. Our study is the first to demonstrate that miR-146a-5p can be horizontally transferred from Mϕs to VSMCs, where it exerts cell-autonomous effects that contribute to disease progression by inducing profound phenotypic and metabolic changes in the recipient cells.

Our data demonstrate that miR-146a-5p is acquired by VSMCs through direct contact with Mϕs, particularly under pro-inflammatory stimuli. Cell-to-cell communication, which can regulate proliferation and differentiation in tissue homeostasis and organ development,^55^ can occur through extracellular vesicles or direct membrane contacts.^55^ Gap junctions—intercellular channels that directly connect the cytoplasm of two cells—allow ions and molecules, including miRNAs, to pass through a regulated gate between cells.^28^ Our findings add to the growing body of evidence that miRNAs can function as paracrine or juxtacrine signalling molecules. Moreover, they highlight the importance of direct cytoplasmic exchange through connexin-based channels, particularly CX43,^29,56^ in mediating miR-146a-5p transfer. This mechanism ensures spatial specificity and rapid signal propagation, especially in the tightly packed cellular environment of the vascular wall. To demonstrate this route of transfer for miR-146a-5p, we developed a short hairpin RNA to induce *Cx43*-specific silencing. Coculture experiments using this *Cx43* inhibition system confirmed that miR-146a-5p transfer was prevented by the absence of the protein.

Traditionally, VSMCs have been classified into two phenotypic states: a contractile, quiescent phenotype and a synthetic, proliferative phenotype.^57^ However, recent lineage-tracing and single-cell transcriptomic studies have demonstrated that VSMCs instead undergo phenotypic modulation along a continuum of context-dependent states while retaining lineage identity.^58–60^ In this contemporary framework, our data suggest that miR-146a-5p promotes a modulated VSMC state characterized by increased proliferation, reduced migration, enhanced contractility, and altered lipid metabolism, rather than inducing a discrete hybrid phenotype. Nonetheless, this behaviour is not totally unexpected, since we are aware that, in some conditions, migration and proliferation are not necessarily interdependent in VSMCs.^61^ Furthermore, in a pro-atherosclerotic context, VSMCs adopt Mϕ-like^62^ and/or foam-like^63^ phenotype transcriptional programmes while remaining within the VSMC lineage, as demonstrated by multiple lineage-tracing studies.^64^ Importantly, accumulating evidence indicates that true transdifferentiation of VSMCs into bona fide Mϕ is exceedingly rare *in vivo*, supporting the interpretation that these states represent modulated VSMC phenotypes rather than lineage conversion.^65^ Our RNA-seq data suggest that miR-146a-5p is, indeed, involved in further VSMC processes, including cholesterol biosynthesis, that are characteristics of modulated VSMC states observed in atherosclerotic lesions. This hypothesis was experimentally confirmed by measuring cholesterol production and expression of Mϕ-associated genes such as *ApoE*, *Thbs1*, and *Lgals3* in VSMCs overexpressing miR-146a-5p. This is particularly relevant in the context of atherosclerosis, where VSMCs contribute not only to plaque stability but also to lipid accumulation and immune cell recruitment. The metabolic reprogramming observed in VSMCs overexpressing miR-146a-5p mirrors changes seen in activated Mϕs and underscores the convergence of inflammatory and metabolic signalling across distinct but interacting cell lineages within the atherosclerotic plaque.

A possible mechanistic explanation of these observations lies in the target of miR-146a-5p we identified, namely *Klf*4. miR-146a-5p transcription is regulated by KLF4 and KLF5, indicating that miR-146a and KLF4 are involved in a feedback loop that is significant for VSMC biology.^66^  *KLF4* is associated with stem cell pluripotency^67^ and is known to be modulated by various miRNAs, including the highly VSMC-enriched miRNAs miR-145^68^ and miR-128.^25^ Furthermore, KLF4 acts as a repressor of VSMC differentiation by inhibiting the expression of genes such as *Acta2*, *Sm22*, and *Myh11*.^43,69^ These effects are partly due to KLF4’s ability to compete with MYOCD, inhibiting SRF from binding CArG box chromatin.^43^ KLF4 can also bind to evolutionarily conserved translational control elements adjacent to CArG boxes in VSMC promoters^70,71^ and interact with Histone Deacetylase 2 to deacetylate Histone H4.^72^ On the other hand, it is well-known that KLF4 also reduces cell proliferation by modulating multiple mechanisms.^73,74^ As a result, overexpression of miR-146a in VSMCs promoted differentiation and proliferation by reducing KLF4 protein, in turn enhancing SRF activity and the transcription of several genes responsible for the phenotype observed in miR-146a-5p-expressing VSMCs.

Our *in vivo* experiments using a VSMC-specific miR-146a-5p sponge system in ApoE^−/−^ mice provide compelling evidence for the pathological relevance of this signalling axis. We demonstrate using this VSMC-specific sponge system that specific inhibition of miR-146a-5p transfer to VSMCs *in vivo* reduces atherosclerosis development in ApoE^−/−^ mice fed a hypercholesterolemic diet; this was accompanied by decreased systemic inflammation and reduced infiltration of immune cells into the vascular wall. These effects were associated with lower circulating levels of CCL20, a chemokine known to mediate leukocyte recruitment^49^ and produced by VSMCs that express miR-146a-5p. Importantly, we also demonstrate that miR-146a-5p is enriched in VSMCs within stenotic regions of human atherosclerotic plaque, providing translational relevance to our findings.

Our findings have broader implications for the understanding of vascular homeostasis and disease. They suggest that miRNA-mediated communication between immune and structural cells is a critical determinant of vascular remodelling and inflammation. This paradigm may extend beyond atherosclerosis to other vascular pathologies, such as restenosis, aneurysm formation, and transplant vasculopathy, where VSMC plasticity and immune cell infiltration play key roles.

Moreover, the identification of miR-146a-5p as a transferable, functional molecule opens new avenues for therapeutic intervention. Targeting miR-146a-5p or its downstream effectors in VSMCs could provide a novel strategy to modulate vascular inflammation and prevent plaque progression. Given the cell-type specificity of our sponge system, such approaches could be designed to minimize off-target effects and preserve the beneficial anti-inflammatory functions of miR-146a-5p in Mϕs.

Translational Perspective The identification of processes activated early during atherosclerosis development could improve disease management. The timing of preventive therapies is crucial to avoid the classical consequences of atherosclerosis, such as myocardial infarction, stroke and claudication. miR-146a-5p is an epigenetic factor associated with inflammation in several diseases, including cardiovascular conditions. Its role in originating cells, like macrophages, is significant and well-studied. We demonstrate that it is transferred to vascular smooth muscle cells, altering their biology and promoting a pro-inflammatory environment in vessels. Inhibiting this transfer reduces plaque progression. Stenotic human plaques exhibit increased expression of miR-146a-5p compared with not-stenotic portions.

## Supplementary Material

cvag075_Supplementary_Data
