# Supplementary material for: miR-146a-5p mediates atherogenic signalling from immune to vascular cells
Source: Cardiovasc Res. 2026 Apr 13;122(8):1022–36. doi: 10.1093/cvr/cvag075 (PMC13241058; doi:10.1093/cvr/cvag075)
Supplement: cvag075_Supplementary_Data [file cvag075_supplementary_data.zip › Climent_et_al_2025_-_De_Novo_-_Supplementary_Data.pdf]

SUPPLEMENTARY DATA

miR-146a-5p mediates atherogenic signaling from immune to vascular cells

Montserrat Climent, PhD, Stefania Zani, PhD, Nicolò Salvarani, PhD, Marco Cremonesi, PhD, Simone Serio, M.Sc, Anna Sbalchiero, M. Sc, Alfonso Tramontano, PhD, Luca Lambroia, PhD, Alice Mallia, M.Sc, Efrem Civilini, MD, Cristina Banfi, PhD, and Leonardo Elia, PhD

Short title: miR-146a-5p signalling from immune to vascular cells

Supplementary Figures and Figure Legends

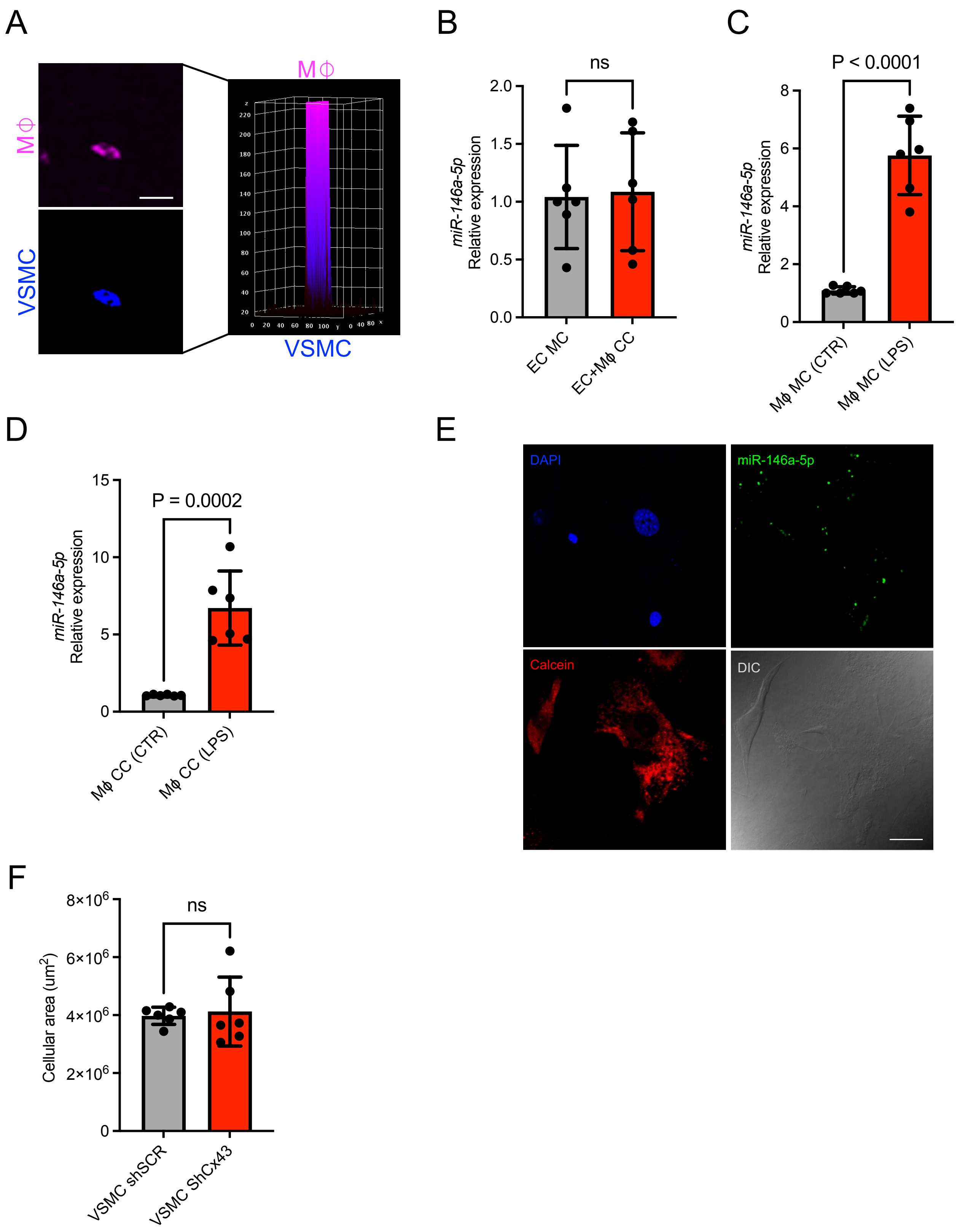

Supplementary Figure 1. miR-146a-5p transfer from Mϕs to ECs and VSMCs. (A) Representative images showing both sides of the PET insert (left) and a reconstructed Z-stack image

(right) of cocultured Calcein Red-labeled Mϕs and Calcein Pacific Blue-labeled VSMCs. Scale bar: 10 µm. **(B)** Mature miR-146a-5p (n=6) was measured in ECs after 36 hours of co-culture with Mϕs or alone (control, CTR). **(C and D)** Mature miR-146a-5p measured by RT-qPCR in un- and LPS-stimulated Mϕs culture alone (n=6) or in co-culture (CC) with VSMCs (n=6). **(E)** Single-channel representative images of the calcein-labeled VSMCs cocultured with Mϕs transduced with the fluorescent miR-146a-5p mimic shown in Figure 1I. Scale bar: 10 µm. **(F)** Area quantification of shSCR and shCx43 VSMCs labeled with phallodin (n=6; at least 30 cells were quantified in each experiment).

For mature miRNA evaluation via RT-qPCR, *U6* snRNA or *mmu-SNORD65* were used as internal controls. Data represent the mean ± SD. To compare means unpaired Student's *t* test was used in A, B, and C. ns = not statistically significant.

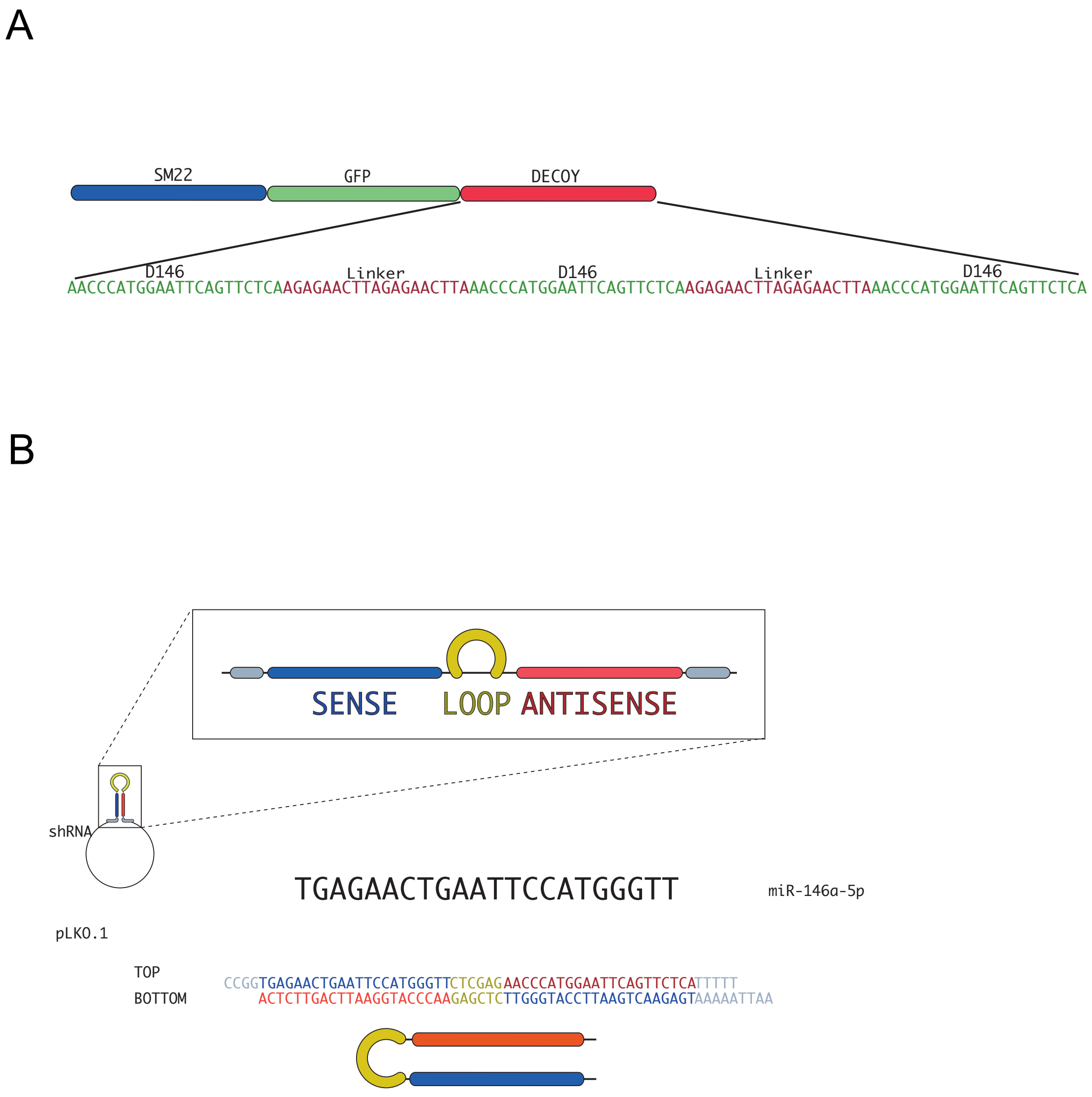

Supplementary Figure 2. Vector design. Schematic representation of the design of the miR-146a-5p decoy (**A**) and overexpressing vectors (**B**).

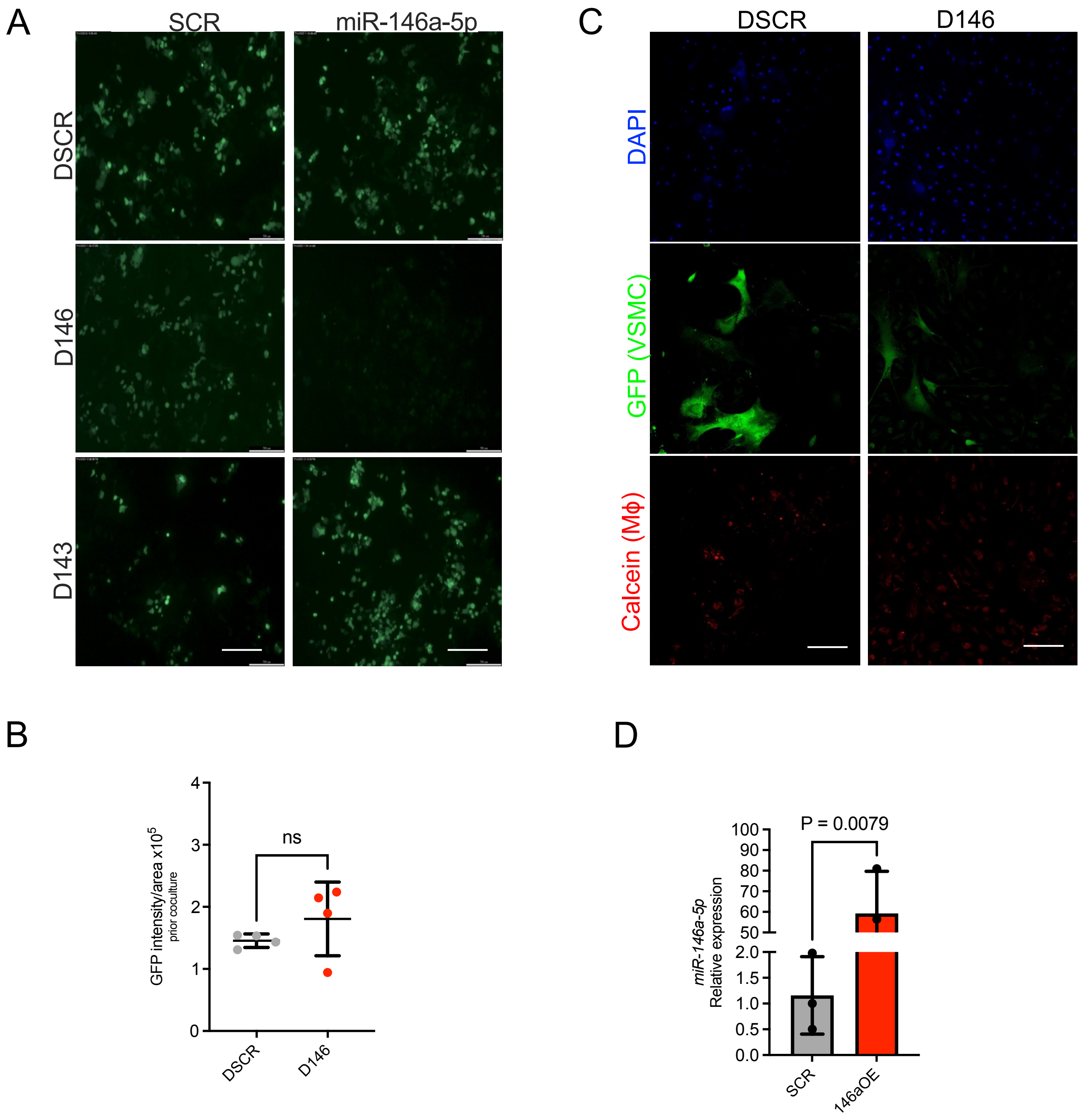

Supplementary Figure 3. D146 functionality *in vitro*. (A) Representative single-channel images of cells expressing sequences of a decoy miR-146a-5p (D146), a decoy miR-143-3p (D143)l, or a scrambled construct (DSCR), and then transduced with a vector carrying miR-146a-5p or a scrambled sequence (SCR). Scale bar: 200 µm. **(B)** Quantification of GFP signal in VSMCs prior to coculture with Mϕs (n=4; at least 15 cells quantified in each experiment). **(C)** Representative single-channel images of VSMCs transduced with a GFP scrambled (DSCR) or miR-146a-5p (D146) decoy sequence, cocultured with calcein-labeled Mϕs for the experiment shown in Figure 2F. **(D)** Mature miR-146a-5p (n=3) was measured via RT-qPCR in VSMCs infected with control virus (SCR) or with the virus generated for the overexpression of the miRNA (146aOE). Scale bar:100 µm.

For mature miRNA evaluation via RT-qPCR, *U6* snRNA or *mmu-SNORD65* were used as internal controls. Data represent the mean ± SD. To compare means, unpaired Student's *t* test was used in B, and D. ns = not statistically significant.

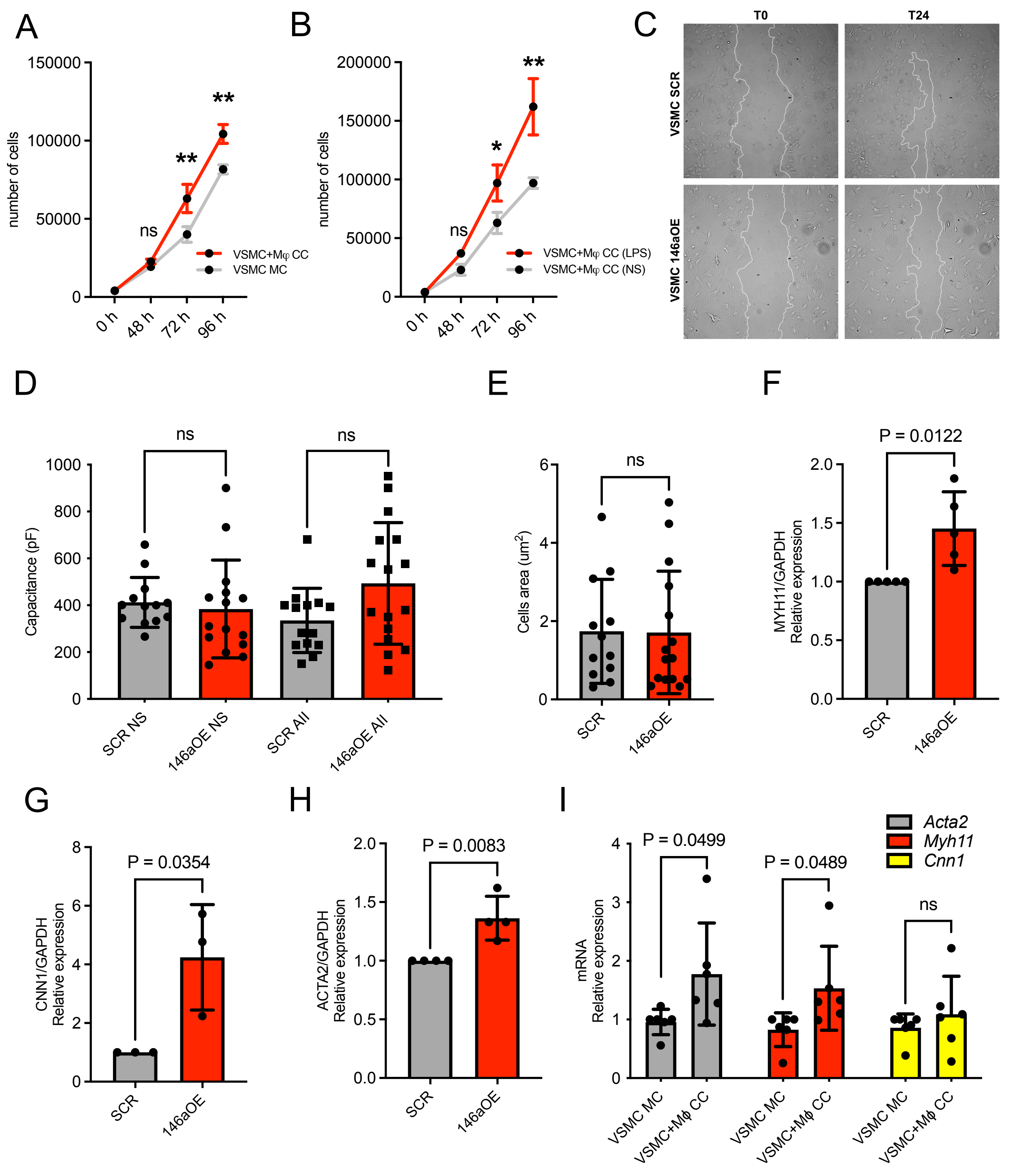

Supplementary Figure 4. miR-146a-5p construct effects on VSMCs. (A) Growth curve of VSMCs in monoculture (MC) or previously cocultured with unstimulated Mϕs (CC; n=3). **(B)** Growth curve of VSMCs previously cocultured with un- (NS) or LPS-stimulated (LPS) Mϕs (n=3). **(C)** Representative image of the scratch assay using SCR and 146aOE VSMCs. **(D)** Capacitance measurements of unstimulated (NS) and AII stimulated (AII) SCR and 146aOE VSMCs. **(E)** Cell area measurements of SCR and 146aOE VSMCs. **(F,H)** Quantification of blots in Figure 3. **(I)** Expression of contraction markers in VSMCs in CC with Mϕs compared to VSMCs in MC (n=6).

Data represent the mean ± SD. To compare means unpaired Student's *t* test was used in A, D, E and F, while 2-way ANOVA with Sidak's multiple comparisons test was used in B. * P=0.0057, ** P<0.0001. ns = not statistically significant.

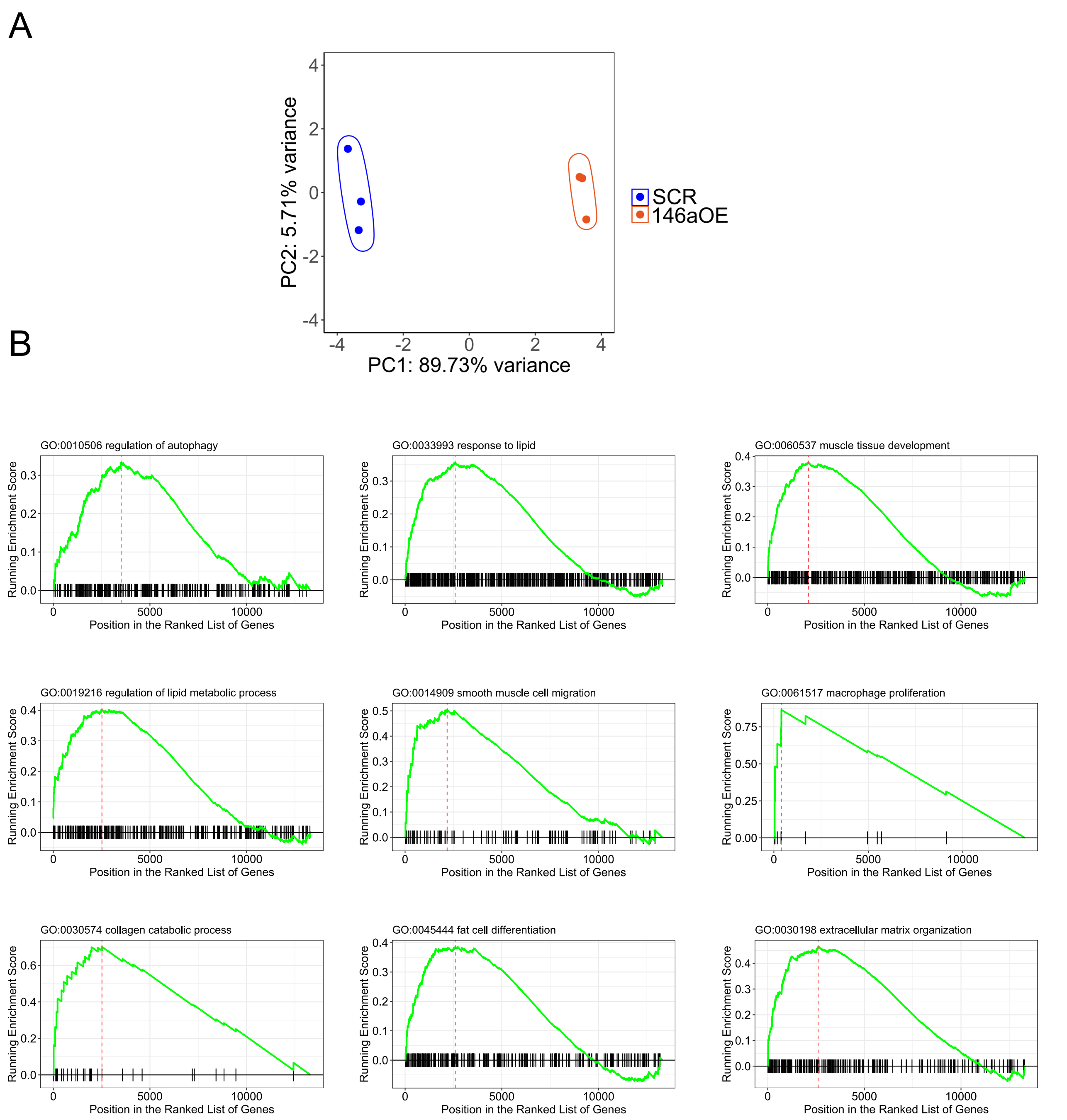

Supplementary Figure 5. Transcriptional profile of miR-146a-5p-overexpressing VSMCs. (A) Principal Component Analysis (PCA) of transcriptional profiles of 146aOE and SRC samples. **(B)** GSEA enrichment plot of enriched terms: regulation of autophagy (GO:0010506), response to lipid (GO:0033993), muscle tissue development (GO:0060537), regulation of lipid metabolic process (GO:0019216), smooth muscle cell migration (GO:0014909), macrophage proliferation (GO:0061517), collagen catabolic process (GO:0030574), fat cell differentiation (0045444) and extracellular matrix organization (GO=0030198).

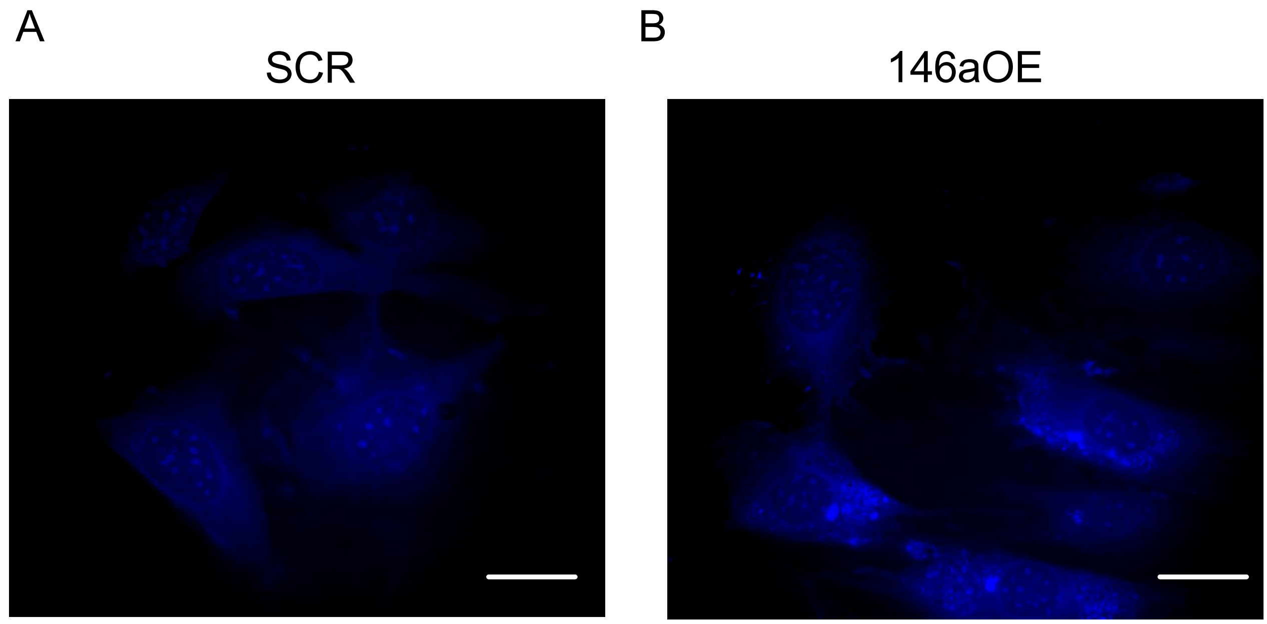

Supplementary Figure 6. Evaluation of internal cholesterol accumulation in miR-146a-5p-overexpressing VSMCs. (A,B) Representative image of Filipin III-labeled SCR **(A)** and 146aOE VSMCs **(B)** for cholesterol. Scale bar:10 μm.

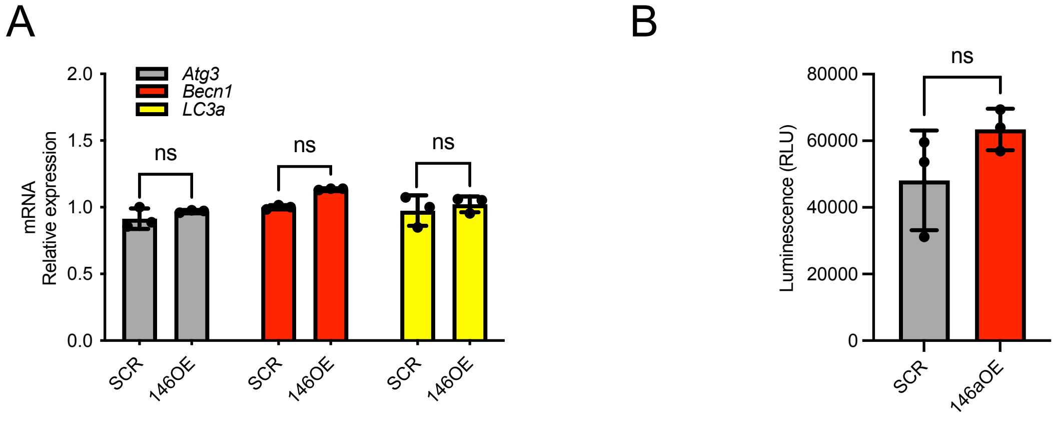

Supplementary Figure 7. Evaluation of autophagy activation in miR-146a-5p-overexpressing VSMCs. (A) Expression of genes involved in autophagy in SCR and 146aOE cells, measured by RT-qPCR (n=3). **(B)** Luminescent signals recorded in scrambled VSMC (SCR) or over-expressing the miRNA (146aOE), using Autophagy LC3 HiBiT Reporter Assay System (Promega).

For gene expression via RT-qPCR, *Ppia* was used as internal control. Data represent the mean ± SD. To compare means unpaired Student's *t* test was used. ns = not statistically significant.

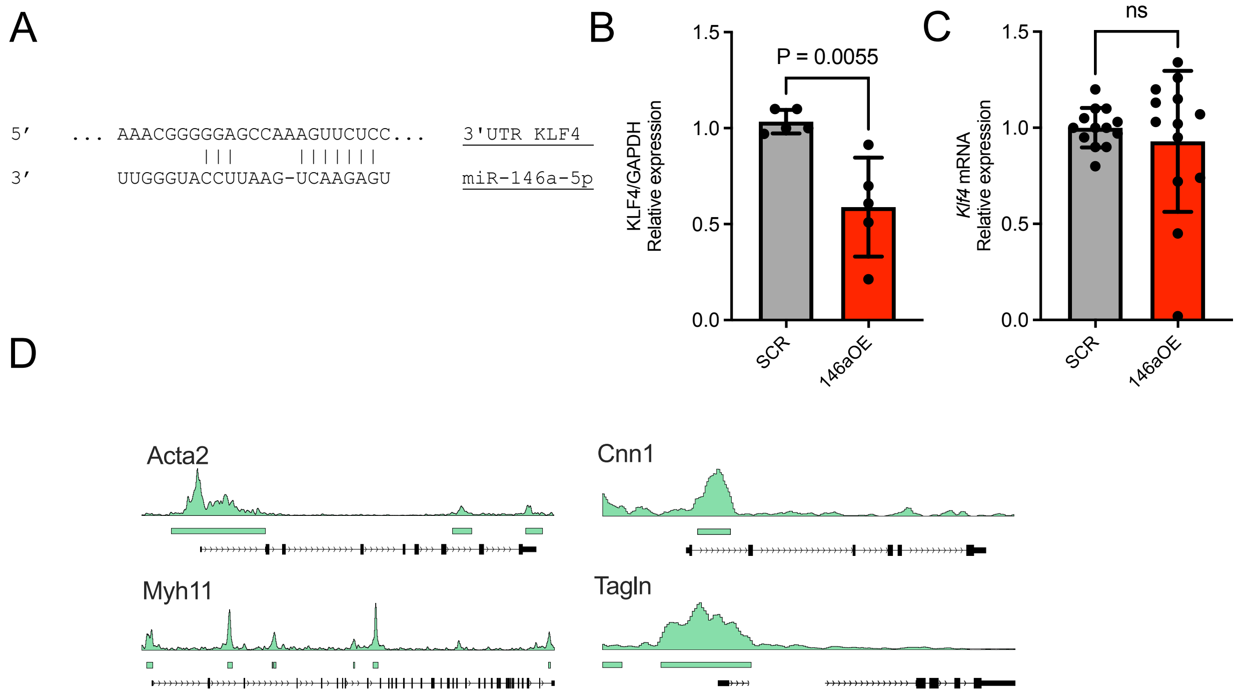

Supplementary Figure 8. miR-146a-5p target identification. (A) Schematic representation of the interaction between miR-146a-5p and 3'UTR *Klf4*. **(B)** Quantification of Western blots shown in Figure 5E (n=5). **(C)** Expression of *Klf4* in SCR and 146aOE cells, measured by RT-qPCR (n=13). **(D)** Enriched SRF ChIP-seq peaks in typical differentiation genes of VSMCs modulated by miR-146a-5p. Horizontal green bars indicate statistically enriched SRF binding sites.

For gene expression analysis via RT-qPCR, *Ppia* was used as internal control. Data represents the mean ± SD. To compare means, unpaired Student's t-test was used in B and C.

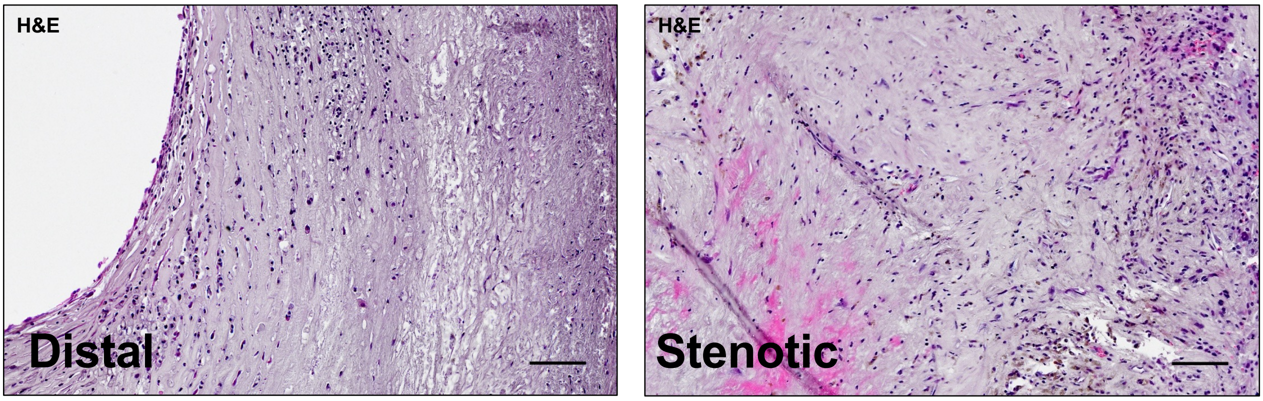

Supplementary Figure 9. Human plaque analysis. Representative H&E images of the distal and stenotic parts of the specimen shown in Figure 7A and 7B. Scale bar: 100 µm.

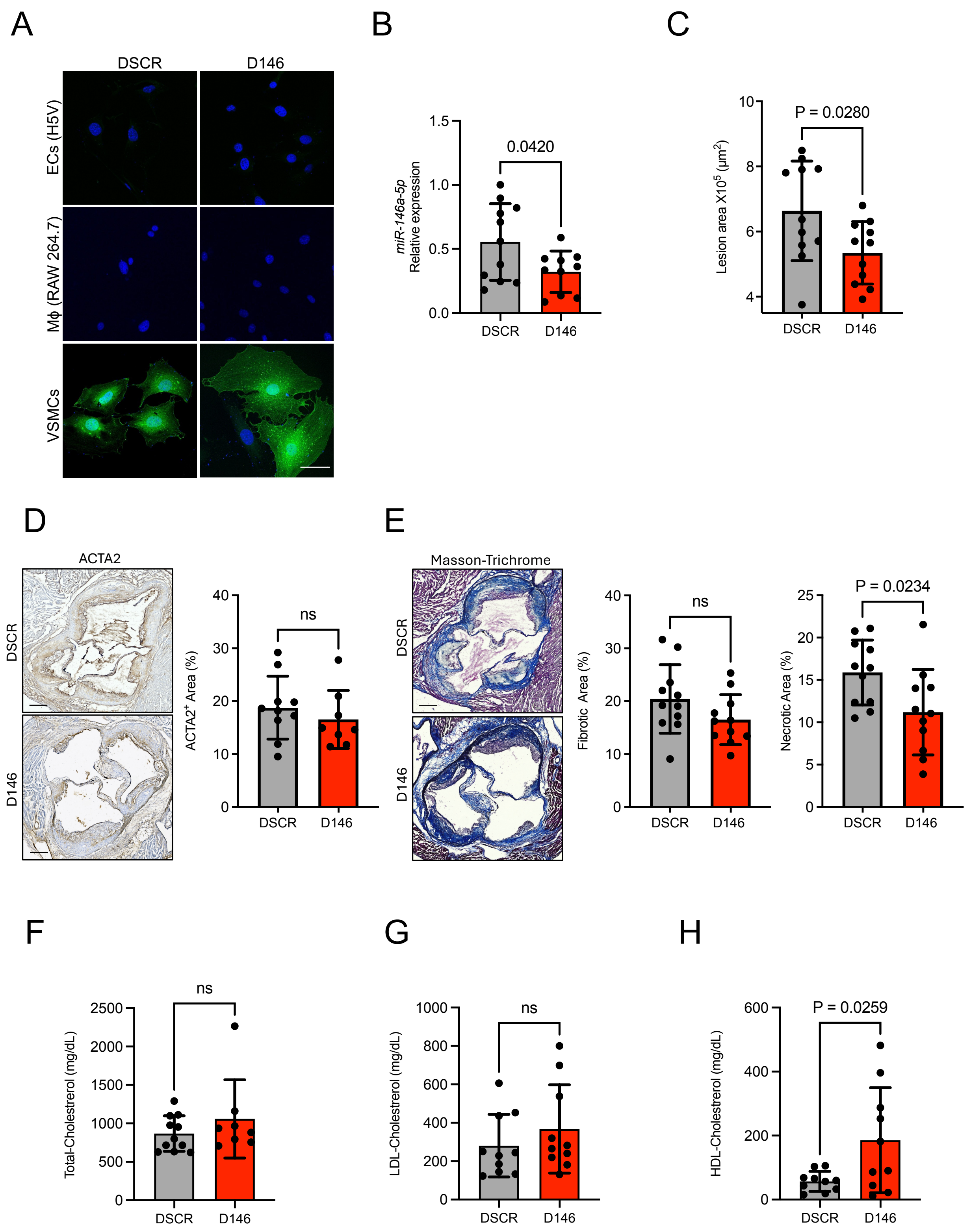

Supplementary Figure 10. Activity of the SM22-D146 system *in vitro* and *in vivo*. (A) Immortalized ECs (H5V), immortalized Mϕs (RAW 264.7), and primary VSMCs were infected with the SM22-GFP decoy constructs (DSCR and D146). Representative images are shown. Scale bar: 40 µm. **(B)** miR-146a-5p measured via RT-qPCR in aortas of ApoE$^{-/-}$ mice transduced with DSCR (n=11) or D146 (n=10) viruses. **(C)** Quantification of lesions shown in Figure 7, considering the total plaque area (DSCR, n=11; D146, n=11). **(D)** Representative ACTA2 staining images of DSCR and

D146 animals and relative quantification of percentage (%) of ACTA2$^+$ area normalized for the total area (DSCR, n=10; D146, n=8). Scale bar: 200 μm **(E)** Representative masson-trichrome staining images of DSCR and D146 animals and quantification of percentage (%) of fibrotic and necrotic areas normalized for the total area (DSCR, n=11; D146, n=11). Scale bar: 200 μm. **(F,H)** Quantification of total (DSCR: n=11; D146: n=8), LDL (DSCR: n=10; D146; n=10), and HDL (DSCR: n=10; D146: n=10) cholesterol in the plasma of ApoE$^{-/-}$ mice transduced with DSCR or D146 viruses.

For mature miRNAs evaluation via RT-qPCR, *U6* snRNA or *mmu-SNORD65* were used as internal controls. Data represent the mean ± SD. To compare means unpaired Student's *t* test was used in B, C, D, E and H, while Mann-Whitney test was used in F and G.

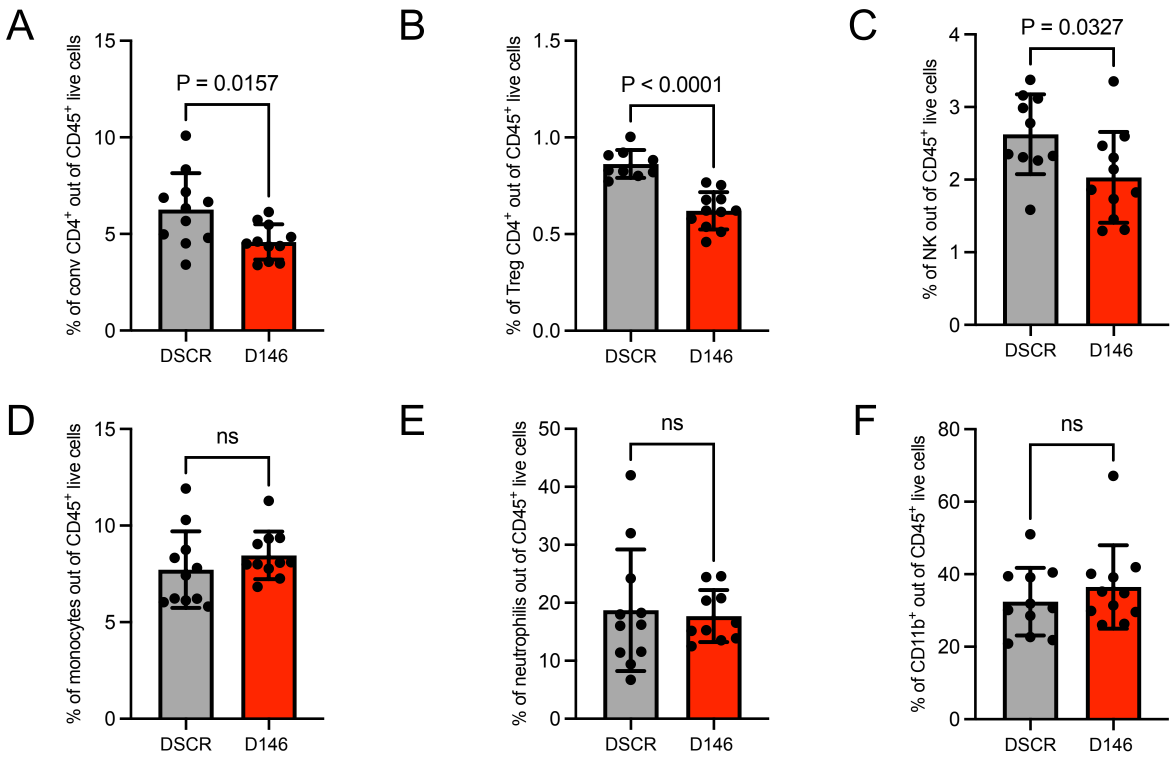

Supplementary Figure 11. *In vivo* effects on circulating cells due to specific miR-146a-5p inhibition in VSMCs during atherosclerosis development. (A-F) FACS analysis of circulating Conv $CD4^+$ T cells **(A)**, Treg $CD4^+$ T cells **(B)**, NK cells **(C)**, monocytes (**D**), neutrophils **(E)**, and $Cd11b^+$ **(F)** cells in DSCR- and D146-treated animals (n=11).

Data represent the mean ± SD. To compare means unpaired Student's *t* test was used. ns = not statistically significant.

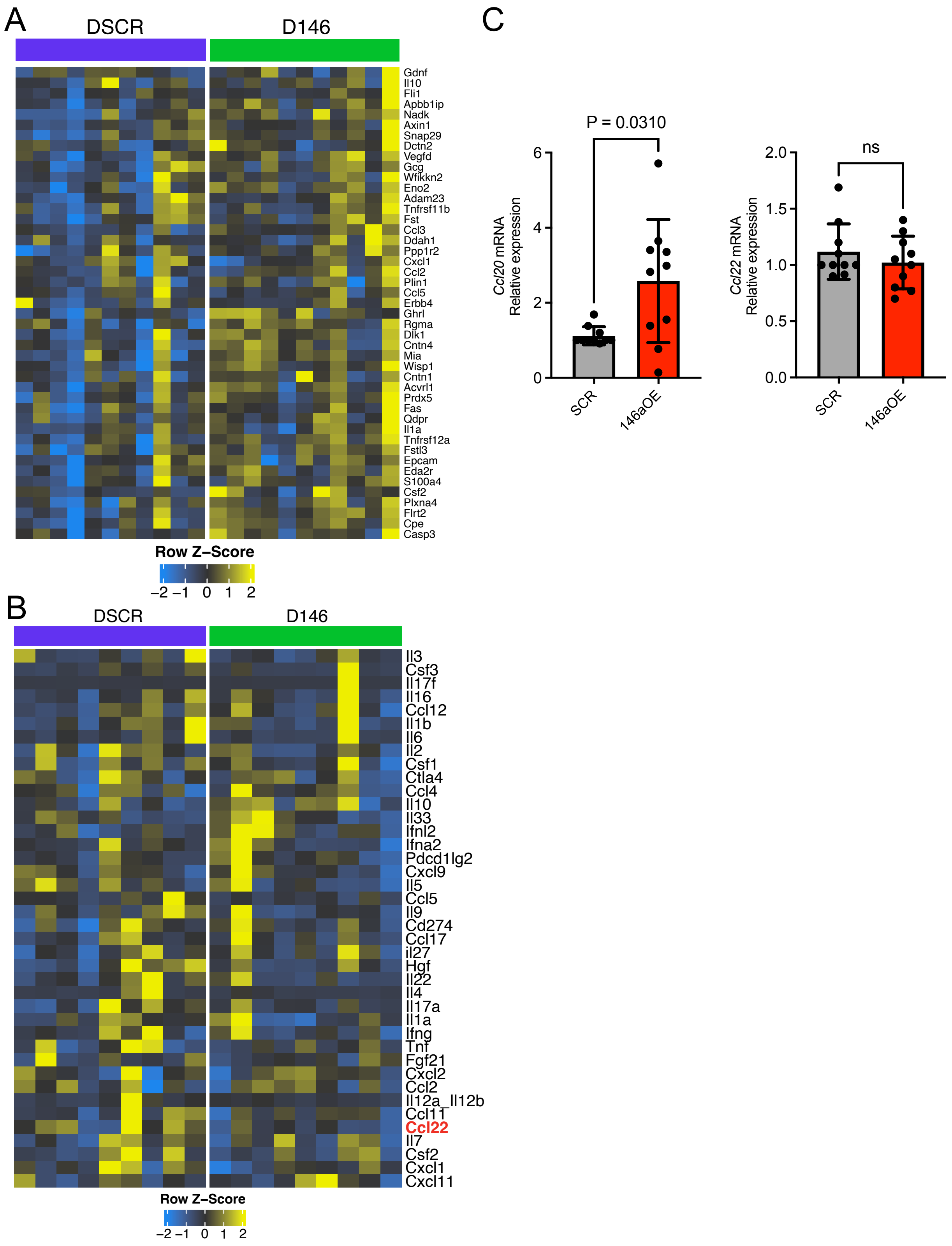

Supplementary Figure 12. ***In vivo*** **effects on secreted factors due to specific miR-146a-5p inhibition in VSMCs during atherosclerosis development. (A)** Analysis of circulating factors in serum of treated animals, using the Olink technology exploratory pannel (n=9) for the cytokines not included in Figure 7G. **(B)** Analysis of circulating factors in serum of treated animals, using the Olink

technology cytokine panel (n=9). **(C)** Relative expression of Ccl20 and Ccl22 in SCR and 146aOE cells, measured by RT-qPCR (n≥3).

For RT-qPCR evaluation, *Ppia* was used as internal control. For ΔΔct analysis, we selected a single reference sample of SCR with a value of 1. Data represents the mean ± SD. To compare means, unpaired Student's t-test was used.

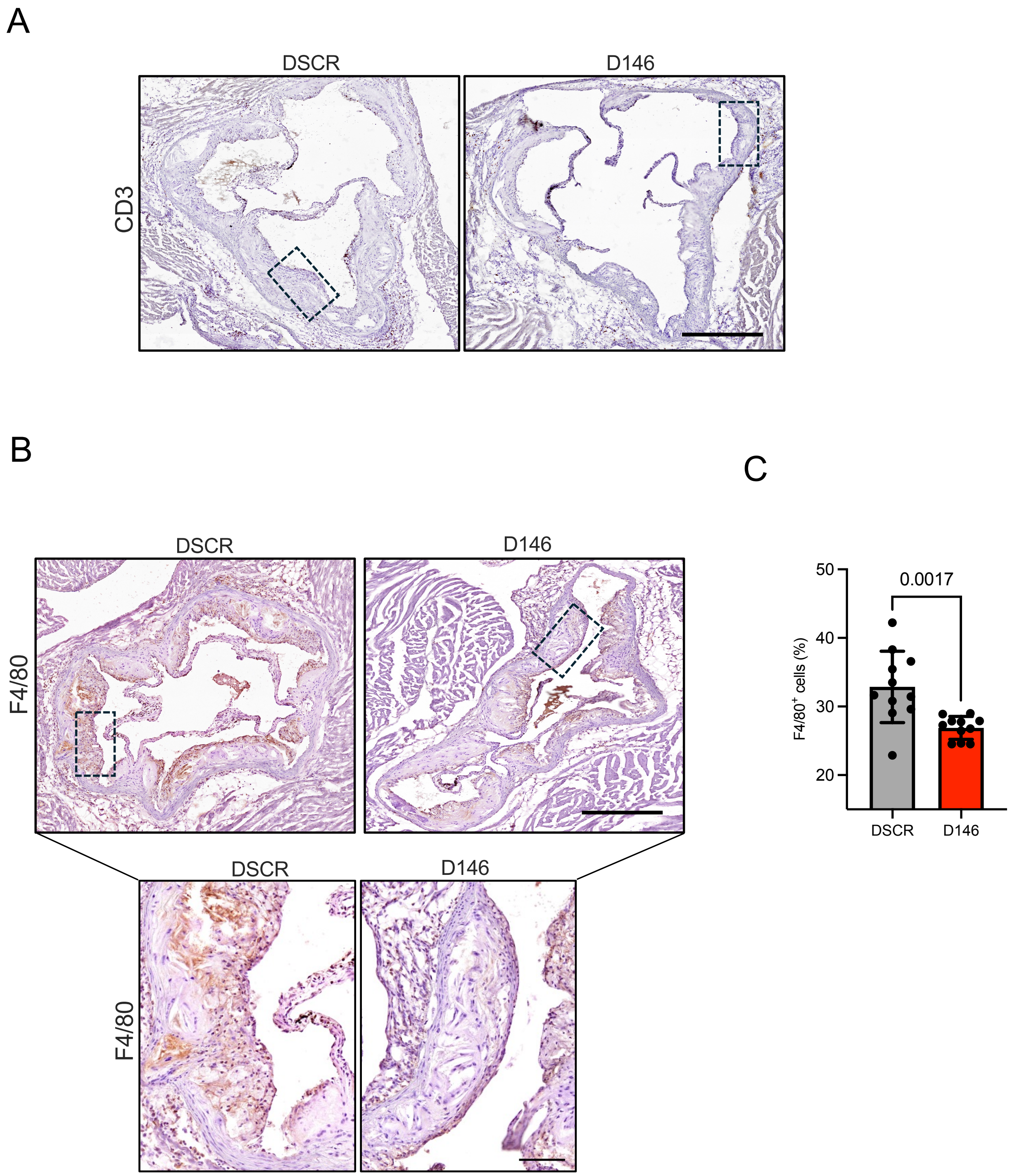

Supplementary Figure 13. *In vivo* effects of specific miR-146a-5p inhibition in VSMCs during atherosclerosis development. (A) Representative CD3 staining images of DSCR and D146 animals, whose magnifications are included in Figure 7H. Scale bar: 500 µm. **(B)** Representative F4/80 staining images of DSCR and D146 animals. Scale bars: 500 and 50 µm **(C)** Quantification of F4/80 positive cells (%) (DSCR, n=11; D146, n=11).

Data represent the mean ± SD. To compare means unpaired Student's *t* test was used. ns = not statistically significant.

Supplementary Tables

Supplementary Table 1. Characteristics of patients analyzed in Figure 6.

Clinical Features	Age	Sex	BMI	Hyp	Dys	Vessel	Max diameter	Symptoms
Patient #1	65	F	25.1	x	x	left ICA	80%	asymptomatic
Patient #2	56	M	27.0	x	x	left ICA	90%	asymptomatic
Patient #3	77	F	24.6	x	x	left ICA	70%	asymptomatic

ICA: Internal carotid
Hyp: Hypertension
Dys: Dyslipidemia

Supplementary Table 2. RT-qPCR primers.

Gene	Forward (5'-3')	Reverse (5'-3')
Ppia	ATTCTGTGAAAGGAGGAACC	CACCGTGTTCTTCGACATC
Pri-mir-146a	GTCTGTGTGTATCCCCAGCT	TCCGTGATGACAGAGCTATCC
Cx43	GGACTGCTTCCTCTCACGTC	GAGCGAGAGACACCAAGGAC
Acta2	CTGACAGAGGCACCACTGAA	CATCTCCAGAGTCCAGCACA
Myh11	GACAACTCCTCTCGCTTTGG	GCTCTCCAAAAGCAGGTCAC
Cnn1	GGCAGGAACATCATTGGACT	GACCTGGCTCAAAGATCTGC
Klf4	CCAAAGAGGGGAAGAAGGTC	CTGTGTGAGTTCGCAGGTGT
Apoe	CTGACAGGATGCCTAGCCG	CGCAGGTAATCCCAGAAGC
Pparg	TTTTCCGAAGAACCATCCGATT	ATGGCATTGTGAGACATCCCC
Hmgcs2	GAAGAGAGCGATGCAGGAAAC	GTCCACATATTGGGCTGGAAA
Thbs1	GGGGAGATAACGGTGTGTTTG	CGGGGATCAGGTTGGCATT
Lgals3	GGAGAGGGAATGATGTTGCCT	TCCTGCTTCGTGTTACACACA
Abca1	GCTTGTTGGCCTCAGTTAAGG	GTAGCTCAGGCGTACAGAGAT
Il6	ACTCCAGAAGACCAGAGGAA	TACCACTCCCAACAGACCTG
Atg3	GAAGAAGATGATGGTGATGGGG	CTTGCTTTCCAGTGTAATCTCCT
Becn1	CTACGCCCAGATCCAGCT	TGATTGTGCCAAACTGTCCG
LC3a	ATCGAGCGCTACAAGGGTG	GGATGATCTTGACCAACTCGC
Myocd	TTCCAAAGATCCCTGGGTCC	CGTTGGCGTAGTGATCGAAG
Ccl20	GCCTCTCGTACATACAGACGC	CCAGTTCTGCTTTGGATCAGC
Ccl22	CTTCTTGCTGTGGCAATTCA	TCGGTTCTTGACGGTTATCA

Supplementary Table 3. Sequences cloned in the pLKO.1 expression vector.

Name	Sequence
shSCR top	CCGGTCCTAAGGTTAAGTCGCCCTCGCTCGAGCGAGGGCGACTTAACCTTAGG TTTTT
shSCR bottom	AATTAAAAATCCTAAGGTTAAGTCGCCCTCGCTCGAGCGAGGGCGACTTAACC TTAGG
shCx43 top	CCGGCCTGATGACCTGGAGATTTAACTCGAGTTAAATCTCCAGGTCATCAGGT TTTT
shCx43 bottom	AATTAAAAACCTGATGACCTGGAGATTTAACTCGAGTTAAATCTCCAGGTCAT CAGG
miR-146a-5p top	CCGGTGAGAACTGAATTCCATGGGTTCTCGAGAACCCATGGAATTCAGTTCTC ATTTTT
miR-146a-5p bottom	AATTAAAAATGAGAACTGAATTCCATGGGTTCTCGAGAACCCATGGAATTCAG TTCTCA

Supplementary Table 4. Decoy sequences cloned in the Tween-SM22 expression vector.

Name	Sequence
DSCR forward	TCGAGAAGGTATATTGCTGTTGACAGTGAGCGCCCTAAGGTTAAGTCGCCCTCGTAGTGAAGCCACAGATGTACGAGGGCGACTTAACCTTAGGTTGCCTACTGCCTCGGCCACGGGG
DSCR reverse	GTGGCCGAGGCAGTAGGCAACCTAAGGTTAAGTCGCCCTCGTACATCTGTGGCTTCACTACGAGGGCGACTTAACCTTAGGGCGCTCACTGTCAACAGCAATATACCTTC
D146 forward	TCGAGAAACCCATGGAATTCAGTTCTCAAGAGAACTTAGAGAACTTAAACCCATGGAATTCAGTTCTCAAGAGAACTTAGAGAACTTAAACCCATGGAATTCAGTTCTCACCACGGGG
D146 reverse	GTGGTGAGAACTGAATTCCATGGGTTTAAGTTCTCTAAGTTCTCTTGAGAACTGAATTCCATGGGTTTAAGTTCTCTAAGTTCTCTTGAGAACTGAATTCCATGGGTTTC
D143 forward	TCGAGAGAGCTACAGTGCTTCATCTCAAGAGAACTTAGAGAACTTAGAGCTACAGTGCTTCATCTCAAGAGAACTTAGAGAACTTAGAGCTACAGTGCTTCATCTCACCACGGGG
D143 reverse	GTGGTGAGATGAAGCACTGTAGCTCTAAGTTCTCTAAGTTCTCTTGAGATGAAGCACTGTAGCTCTAAGTTCTCTAAGTTCTCTTGAGATGAAGCACTGTAGCTCTC

Supplementary Table 5. Olink Target 96 Mouse Exploratory panel

UniProt ID	Gene	Protein name
P20826	KITLG	Kit ligand
Q9D6N1	CA13	Carbonic anhydrase 13
P47713	PLA2G4A	Cytosolic phospholipase A2
P10855	CCL3	C-C motif chemokine 3
P48787	TNNI3	Troponin I, cardiac muscle
Q04736	YES1	Tyrosine-protein kinase Yes
Q00493	CPE	Carboxypeptidase E
P12960	CNTN1	Contactin-1
Q9JLL0	CRIM1	Cysteine-rich motor neuron 1 protein
Q8R5A3	APBB1IP	Amyloid beta A4 precursor protein-binding family B member 1-interacting protein
Q61865	MIA	Melanoma-derived growth regulatory protein
P25446	FAS	Tumor necrosis factor receptor superfamily member 6
Q8VCF1	CANT1	Soluble calcium-activated nucleotidase 1
Q99JW5	EPCAM	Epithelial cell adhesion molecule
Q8CD15	RIOX2	Ribosomal oxygenase 2
P48540	GDNF	Glial cell line-derived neurotrophic factor
Q6PCX7	RGMA	Repulsive guidance molecule A
P31240	PDGFB	Platelet-derived growth factor subunit B
P08505	IL6	Interleukin-6
P47931	FST	Follistatin
O35625	AXIN1	Axin-1
Q62386	IL17A	Interleukin-17A
P07321	EPO	Erythropoietin
P58058	NADK	NAD kinase
Q9R1E0	FOXO1	Forkhead box protein O1
Q80UG2	PLXNA4	Plexin-A4
Q8CGN5	PLIN1	Perilipin-1
Q7TQN3	WFIKKN2	WAP, Kazal, immunoglobulin, Kunitz and NTR domain-containing protein 2
P10148	CCL2	C-C motif chemokine 2
P30561	AHR	Aryl hydrocarbon receptor
P18406	CCN1	CCN family member 1
Q8BLU0	FLRT2	Leucine-rich repeat transmembrane protein FLRT2
Q9Z0T9	ITGB6	Integrin beta-6
Q9R1V7	ADAM23	Disintegrin and metalloproteinase domain-containing protein 23
P97785	GFRA1	GDNF family receptor alpha-1
Q9DCL8	PPP1R2	Protein phosphatase inhibitor 2
Q8BVI4	QDPR	Dihydropteridine reductase
Q09163	DLK1	Protein delta homolog 1
P17183	ENO2	Gamma-enolase
Q8R373	CLMP	CXADR-like membrane protein
P30882	CCL5	C-C motif chemokine 5
O08712	TNFRSF11B	Tumor necrosis factor receptor superfamily member 11B
P04202	TGFB1	Transforming growth factor beta-1 proprotein
P48030	TGFA	Protransforming growth factor alpha

P99029	PRDX5	Peroxiredoxin-5, mitochondrial
O08746	MATN2	Matrilin-2
Q61982	NOTCH3	Neurogenic locus notch homolog protein 3
Q61527	ERBB4	Receptor tyrosine-protein kinase erbB-4
P55095	GCG	Pro-glucagon
Q9ER65	CLSTN2	Calsyntenin-2
P07091	S100A4	Protein S100-A4
Q9ERB0	SNAP29	Synaptosomal-associated protein 29
P01582	IL1A	Interleukin-1 alpha
Q4V9Z5	SEZ6L2	Seizure 6-like protein 2
Q8K4B4	IL23R	Interleukin-23 receptor
Q61483	DLL1	Delta-like protein 1
Q9CWS0	DDAH1	N(G),N(G)-dimethylarginine dimethylaminohydrolase 1
Q08048	HGF	Hepatocyte growth factor
Q8BTW9	PAK4	Serine/threonine-protein kinase PAK 4
Q9CR75	TNFRSF12A	Tumor necrosis factor receptor superfamily member 12A
P12850	CXCL1	Growth-regulated alpha protein
P18893	IL10	Interleukin-10
P97326	CDH6	Cadherin-6
Q61288	ACVRL1	Serine/threonine-protein kinase receptor R3
O89017	LGMN	Legumain
O88393	TGFBR3	Transforming growth factor beta receptor type 3
Q6ZQA6	IGSF3	Immunoglobulin superfamily member 3
P01587	CSF2	Granulocyte-macrophage colony-stimulating factor
P70236	MAP2K6	Dual specificity mitogen-activated protein kinase kinase 6
Q9R000	ITGB1BP2	Integrin beta-1-binding protein 2
Q7TNI7	IL17F	Interleukin-17F
P10749	IL1B	Interleukin-1 beta
P70677	CASP3	Caspase-3
O54775	CCN4	CCN family member 4
P18340	CXCL9	C-X-C motif chemokine 9
Q9Z109	VSIG2	V-set and immunoglobulin domain-containing protein 2
P04401	IL5	Interleukin-5
P26323	FLI1	Friend leukemia integration 1 transcription factor
O89093	CCL20	C-C motif chemokine 20
Q8BYI9	TNR	Tenascin-R
O54907	TNFSF12	Tumor necrosis factor ligand superfamily member 12
P20181	NTF3	Neurotrophin-3
P97946	VEGFD	Vascular endothelial growth factor D
Q8BX35	EDA2R	Tumor necrosis factor receptor superfamily member 27
Q99KJ8	DCTN2	Dynactin subunit 2
P11103	Parp1	Poly [ADP-ribose] polymerase 1
Q9EQC7	Fstl3	Follistatin-related protein 3
P11152	Lpl	Lipoprotein lipase
P06804	Tnf	Tumor necrosis factor
O89023	Tpp1	Tripeptidyl-peptidase 1
Q69Z26	Cntn4	Contactin-4
Q9EQX0	Ghrl	Appetite-regulating hormone

Supplementary Table 6. Olink Target 48 Mouse cytokine panel

UniProt ID	Gene	Protein name
Q14116	IL18	Interleukin-18
P14210	HGF	Hepatocyte growth factor
Q99731	CCL19	C-C motif chemokine 19
P13500	CCL2	C-C motif chemokine 2
P39900	MMP12	Macrophage metalloelastase
P01374	LTA	Lymphotoxin-alpha
P49771	FLT3LG	Fms-related tyrosine kinase 3 ligand
P01375	TNF	Tumor necrosis factor
Q16552	IL17A	Interleukin-17A
P60568	IL2	Interleukin-2
Q96PD4	IL17F	Interleukin-17F
P09919	CSF3	Granulocyte colony-stimulating factor
P01584	IL1B	Interleukin-1 beta
P78380	OLR1	Oxidized low-density lipoprotein receptor 1
O43508	TNFSF12	Tumor necrosis factor ligand superfamily member 12
P02778	CXCL10	C-X-C motif chemokine 10
P15692	VEGFA	Vascular endothelial growth factor A
O95760	IL33	Interleukin-33
Q969D9	TSLP	Thymic stromal lymphopoietin
P01579	IFNG	Interferon gamma
P13236	CCL4	C-C motif chemokine 4
P01135	TGFA	Protransforming growth factor alpha
P35225	IL13	Interleukin-13
P10145	CXCL8	Interleukin-8
P80075	CCL8	C-C motif chemokine 8
P05231	IL6	Interleukin-6
Q99616	CCL13	C-C motif chemokine 13
P04141	CSF2	Granulocyte-macrophage colony-stimulating factor
P80098	CCL7	C-C motif chemokine 7
P05112	IL4	Interleukin-4
P50591	TNFSF10	Tumor necrosis factor ligand superfamily member 10
P13725	OSM	Oncostatin-M
P03956	MMP1	Interstitial collagenase
P01133	EGF	Pro-epidermal growth factor
P13232	IL7	Interleukin-7
P40933	IL15	Interleukin-15
P09603	CSF1	Macrophage colony-stimulating factor 1
Q07325	CXCL9	C-X-C motif chemokine 9
O14625	CXCL11	C-X-C motif chemokine 11
Q9P0M4	IL17C	Interleukin-17C
P48061	CXCL12	Stromal cell-derived factor 1
P51671	CCL11	Eotaxin
P22301	IL10	Interleukin-10
P10147	CCL3	C-C motif chemokine 3
Q14213_Q8NEV9	EBI3_IL27	Interleukin-27

SUPPLEMENTARY METHODS and REFERENCES

Supplementary Methods

Isolation and cell culture

Primary VSMCs were generated from aorta of C57BL/6J mice (Charles River). After surgical removal, adventitia and intima layers were surgically separated from the media, which was subsequently digested at 37 °C for 1 h with 1 mg/ml collagenase type II (Ca. #CLS-2, Worthington) dissolved in PluriSTEM Dispase-II Solution (Ca. #SCM133, Merck). Digestion was blocked by adding DMEM supplemented with 10 % FBS. Cells were plated on plastic dishes pre-coated with 0.1 % gelatin. Thereafter, VSMCs were characterized by qPCR and immunofluorescence analysis for the expression of specific markers, such as smooth muscle actin (Ca. #A5228, Sigma-Aldrich) and transgelin (Ca. #ab14106, Abcam). The cells were cultured in DMEM supplemented with 10% FBS, 4 mM Glutamine stable (Ca. #ECB3004D, Euroclone), 1 mM Sodium Pyruvate (Ca. #ECM0542D, Euroclone), Penicillin-Streptomycin (Ca. #ECB3001D, Euroclone) and maintained in a humidified incubator at a controlled temperature of 37 °C (5 % CO_2).

Primary Mϕs were generated from the bone marrow of tibia and femur of C57BL/6J mice (Charles River). After surgical removal and bone cleaning from muscle and connective tissues, the ends of tibia and femur were cut in order to harvest the bone marrow via flushing. The next step was cell centrifugation (1500 rpm for 10 min) and supernatant removal. Then, 1 ml of ACK Lysing Buffer (Ca. #A1049201, ThermoFisher Scientific) was added to the pellet for the lysis of red blood cells. After making up to volume with saline solution, the centrifugation step was repeated (1500 rpm for 5 min) to resuspend the pellet in complete IMDM (supplemented with 10 % FBS, 2 mM Ultraglutamine-1 and Penicillin-Streptomycin). The cell suspension was filtered using 70 µm nylon strainer (Falcon). At this point it was possible to determine the yield and viability of cells by counting in Bürker Chambers using Trypan blue. The bone marrow-derived cells obtained were plated in complete IMDM and incubated at 37 °C with 5 % CO_2 in a humidified incubator. The next day, the cells in suspension (monocytes) were collected, centrifuged (1500 rpm for 5 min) and the cells

resuspended in complete IMDM supplemented with Mouse M-CSF (Ca. #130-094-129, Miltenyi Biotec) to the final concentration of 50 ng/ml. After an additional 72 h of culture, the medium was replaced with fresh complete IMDM supplemented with Mouse M-CSF, recovering the monocytes not yet differentiated into Mϕs. At day 7 from the start of the procedure, Mϕs resulted to be adherent to the plate and completely differentiated.

For some experiments, we used a cell line of endothelial cells (H5V)[1] and a cell line of mouse Mϕs (RAW 264.7; Ca. #TIB-71, ATCC) that we maintained in DMEM supplemented with 10 % FBS, 4 mM Ultraglutamine-1 (Lonza), 1 mM Sodium Pyruvate (Euroclone) and antibiotics.

Cell stimulation

For the induction of pro-inflammatory (M1) and anti-inflammatory (M2) phenotypes in primary Mϕs, we stimulated cells with lipopolysaccharide (LPS, 100 ng/ml; Ca. #L2654, Merck) or interleukin-4 (Il-4, 20 ng/ml; Ca. #130-097-757, Miltenyi Biotec) respectively. Cells were maintained in IMDM complete medium with the stimulus for 48 h before collection for RNA extraction.

Functional contraction study

The contractility assay was performed as previously described.[1] Primary VSMCs were detached using trypsin-EDTA (Euroclone), and resuspended at the concentration of 100,000 cells/ml. Then 0.4 ml of cell suspension were mixed to 0.2 ml of 3 mg/ml collagen solution (Ca. #08-115 Sigma) diluted in 0.1 % Acetic Acid. After adding an appropriate volume of 1 M NaOH to the mixture of cells and collagen, 500 μl of the mixture was immediately transferred to a 24-well plate. Gels were allowed to solidify for 20 min at room temperature, and then 0.5 ml of fresh complete DMEM was added. Plates were then transferred into a 37 °C incubator with a humidified 5 % CO_2 atmosphere. The extent of gel contraction was measured by calculating the area of gel with the Image J software.

Luciferase Reporter assay

To test *Klf4* as a target of miR146a-5p, we transfected primary VSMCs using Lipofectamine LTX+Plus reagent (Ca. #15338100, Life Technologies) in 24-well dishes with 500 ng of psiCheck2 reporter vector (Ca. #C8021, Promega) containing *Klf4*, a mutated *Klf4* or *Traf3* 3'UTR region, together with 20 nM miR-146a-5p mimic (Ca. #YM00472124-ADA, Qiagen) or scrambled (SCR) mimic (Ca. #YM00479902-ADA, Qiagen). Luciferase assay was then performed 72 h post-transfection using Dual-Glo Luciferase Assay System as described by the manufacturer (Ca. #E1910, Promega).

To test SRF activity in miR-146a-overexpressing cells, we transfected primary VSMCs using Lipofectamine LTX+Plus reagent (Ca. #15338100, Life Technologies) in 24-well dishes with 500 ng of pGL3-basic ACTA2 luciferase, together with 100 ng of a plasmid carrying the renilla gene, used as internal control, plus 20 nM miR-146a-5p (Ca. #339173-YM00472124, Qiagen), miR-9 (Ca. #YM00471434, Qiagen) and miR-214 (Ca. #339173-YM00472980, Qiagen) mimics or scrambled (Ca. #339173-YM00479902, Qiagen) mimic. Then, renilla and firefly luciferase activity was detected using Dual-Glo Luciferase Assay System 72 h post-transfection.

RNA extraction

MiRNA isolation for small RNA sequencing was performed using Direct-zol RNA Miniprep kit (Ca. #R2050, Zymo Research) according to the manufacturer's protocol. Total RNA was isolated using Nucleozol reagent (Ca. #FC1740404, Macherey-Nagel) according to the manufacturer's protocol. For gene expression, RNA was reverse transcribed with High-Capacity cDNA Reverse Transcription Kit (Ca. #4368813, Applied Biosystems) and qPCR performed with GoTaq SYBR Green (Ca. #A6002, Promega), using *Ppia* as housekeeping genes. PCR primers are listed in Supplementary Table 2. For miRNA expression, RNA was reverse transcribed using miRCURY LNA RT Kit (Ca. #339340, Qiagen). SYBR Green was used to perform qPCR for mmu-miR-146a-5p (Ca. #YP00204688, Qiagen) and U6 snRNA (Ca. #YCP0050862, Qiagen) or mmu-SNORD65 (Ca. #YP00203910, Qiagen) as housekeeping genes. Results are expressed as fold induction (2-ΔΔCt).

Generation of lentiviral vectors and cell infection

Short hairpin RNA for scrambled and Connexin 43 (Cx43) were generated by cloning target sequences in the lentiviral backbone plasmid pLKO.1. To obtain miRNA overexpression we used the same technique but cloning the mature miRNA sequence and its reverse complementary inside of pLKO.1. Sequences cloned in pLKO.1 expression vector are listed in Supplementary Table 3. The cloning process was carried out according to the Addgene's pLKO.1 protocol (https://www.addgene.org/protocols/plko/). To generate a silencing system specific for VSMCs, we cloned decoy sequences (including three consecutive complementary sequences for the miRNAs) for miR-146a-5p (D146), miR-143-3p (D143) or scrambled control (DSCR) after the GFP of a TWEEN-SM22 DECOY expression vector, whose activity is selectively targeted to VSMCs due to the presence of the specific SM22 promoter (Supplementary Table 4).[2] Lentiviral particles (LVPs) were generated in HEK293T cells (Ca. #ATCC-CRL-1573.3, ATCC). Briefly, HEK293T cells were treated with chloroquine-containing media and subsequently transfected with the cloned plasmids and pCMV-VSV-G/pCMV-dR8.2 packaging plasmids using a standard $CaCl_2$ transfection protocol. After 6 h, the transfection medium was removed and replaced with standard 10 % FBS DMEM. 48 and 72 h later, the LVP-containing medium was collected and filtered. Filtered LVP medium was further supplemented with 4M NaCl, 1X PBS, and 50 % PEG for the lentivirus purification procedure. After incubation at 4 °C overnight with gentle swirling, the LVP-containing medium was centrifuged at 1500 RCF for 30 min at 4 °C, resuspended in 1X HBSS, aliquoted, and stored at -80 °C. For the infection of cells with LVPs we use a standard protocol: cells are plated at a 40 % confluence. The next day, infection is carried out in complete DMEM medium supplemented with hexadimethrine bromide (Ca. #H9268, Merck) at a final concentration of $5 \cdot 10^{-3}$ mg/ml. After 48 hours, VSMCs were selected with $5 \cdot 10^{-3}$ mg/ml puromycin dihydrochloride (Ca. #sc-108071B, Santa Cruz Biotechnology) for another 48 h. Once the selection phase is complete, the medium can be replaced with a complete DMEM without puromycin dihydrochloride to proceed with the experiments.

Proliferation assay

To test miR-146a-5p's effect on cell proliferation, we plated 5,000/well primary VSMCs stably expressing miR-146a-5p or a scrambled sequence in 24-well dishes. Moreover, to mimic a more physiological condition, we performed a proliferation assay on primary VSMCs derived from co-cultures with primary Mϕs previously stimulated with LPS (100 ng/ml) or not stimulated (NS) as control. In this case, we plated 4,000 cells per well, using 24-well dishes. After established time points, cells were detached with trypsin-EDTA (Euroclone) and counted in Bürker chamber using Trypan blue to determine the vital cell number.

Migration assay

Cells were cultivated in 12-well plates until they reached 100 % confluence. The wound/scratch on the cell layer was created with a 10 µl pipette tip. Afterwards, cells were washed with 1X PBS and cell migration evaluated over time using a DMI8 Live Cell system (Leica).

Patch-clamp

Patch-clamp experiments were performed on primary VSMCs (P8-9) stably expressing miR-146a-5p or a scrambled sequence. Electrophysiological recordings were performed using a Multiclamp 700B patch-clamp amplifier (Molecular Devices) controlled by pClamp 10.3 software (Molecular Devices). 10,000 cells were seeded on 22 cm glass coverslip (VWR International). 2/3 days after seeding, cells were transferred to a custom-made experimental chamber that was fixed to the stage of an inverted microscope (Nikon eclipse, Ti/U). Recordings were made on cells maintained in basal conditions (not stimulated, NS) or in presence of Angiotensin II (Merck) as a contractile stimulus, at the final concentration of 100 nM. Angiotensin II was added to VSMCs cultures two hours before patch-clamp recordings. Preparations were superfused with Hanks' balanced salt solution (HBSS, Sigma-Aldrich) containing (in mmol/l): NaCl 137, KCl 5.4, CaCl2 1.3, MgSO4 1.2, NaHCO3 4.0, KH2PO4 0.5, NaH2PO4 0.3, D-glucose 5.5 and HEPES 10 (pH 7.40 with KOH) and superfused at 1.5 ml/min. Series resistance and, after rupturing of the patch, cell capacitance were compensated and voltage values were corrected for liquid junction potentials (-12.4 mV). Patch pipettes were pulled

from borosilicate glass capillaries (Intrafil-10, INTRACEL LTD) with a laser-based micropipette puller (P-2000; Sutter Instrument) and resistances ranged from 2 to 4 MΩ. Seal resistances were 2–10 GΩ and rupturing the cell membrane in the patch resulted in access resistances of 2–10 MΩ. Single VSMCs were current-clamped in the whole-cell configuration of the ruptured patch-clamp technique. Membrane potential signals were low-pass filtered at 3 kHz, digitized at 50 kHz and stored for offline analysis. Experimental data analysis was performed using dedicated software (pClamp 10.6 and Clampfit 10.6; Molecular Devices). Vm of VSMCs was assessed at 36 °C in sparsely seeded cell cultures and measured in current-clamp modality, applying 10 seconds long current stimuli with the current value set to zero (pA = 0). Data were obtained from at least 3 different cultures and statistical comparisons were made using Student's t test for unpaired samples. Differences with at least $P < 0.05$ were regarded as significant.

Autophagy LC3 HiBiT Reporter Assay System

To test autophagic capacity, primary VSMCs were transfected with 50 ng of Autophagy LC3 HiBiT Reporter Vector (Ca. #GA255A, Promega), and 750 ng pLKO.1-SCR or pLKO.1-146s vector using Lipofectamine LTX+Plus reagent (Ca. #15338100, Life Technologies). 72h post-transfection, a suspension of 8,000 cells (in 80 µl) was plated in a 96-wells bottom flat plates (Ca. #353072, Falcon). Each condition was tested in duplicate. After allowing the cells to attach overnight in a 37 °C, 5 % CO_2 incubator, the assay was performed as described by the manufacturer (Ca. # GA2551, Promega). Briefly, following 10 min of equilibration at room temperature, one volume of Nano-Glo HiBiT Lytic Reagent was added to each well and the plate then placed on an orbital shaker for 10 min. Cell lysis was promoted by pipetting and lysed transferred in a white 96-well plate to proceed with the measuring at GloMax Discover System (Promega).

Western blotting

5 µg of proteins were loaded into precast polyacrylamide gels (Bolt Bis-Tris Plus Mini Protein Gels 4-12%; Ca. #NW04120BOX, Life Technologies) and run was performed using NuPAGE MOPS SDS

Running Buffer (Ca. #NP0001, Life Technologies), according to the manufacturer's indications. Electrophoretic Transfer (60 minutes, 100 V) was used to transfer proteins from gel to nitrocellulose membrane. Blot blocking was performed with 5% milk in PBS plus 0.05% Tween20 detergent, for 1 h at room temperature with gentle agitation. The primary antibodies were the following: anti-MYH11 (1:1000 dilution; Ca. #21404-1-AP, Protein Tech), anti-CNN1 (1:500 dilution; Ca. #sc-28545, Santa Cruz), anti-ACTA2 (1:1000 dilution; Ca. #A5228, Sigma-Aldrich), anti-GAPDH (1:1000 dilution; Ca. #sc-32233, Santa Cruz), anti-KLF4 (1:1000 dilution; Ca. #ABS1514, Millipore). Incubation with species-specific secondary antibodies was performed for 1 h at room temperature, at a dilution of 1:5000. Development was performed with Immobilon Western Chemiluminescent HRP Substrate (Ca. #WBKLS0500, Millipore).

Cells size analysis

Primary VSMCs over-expressing miR-146a-5p or a scrambled control sequence were fixed with 4% PFA and permeabilized with 0.2% Triton X-100 in PBS for 7 min at room temperature before incubation with Alexa Fluor 594 phalloidin (Ca. #A12381, Life Technologies) diluted 1:200 in a PBS solution containing 1% BSA, for 45 min at room temperature. Then, nuclei were stained with DAPI (Ca. #D1306, Life Technologies) and finally the slides were mounted with ProLong Diamond Antifade Mountant (Ca. #P36961, Life Technologies). Image acquisitions were performed using Confocal Microscopy (SP8, Leica) and the size of the cells were measured with Image J software.

Cholesterol quantification

Sample preparation for mass spectrometry (MS): VSMC samples were extracted using a quenching solution of 2:2:1 (v/v/v) ACN/MeOH/H_2O, then frozen and lyophilized overnight. Prior solubilization, deuterium-labeled cholesterol (Ca. #700172P, Merck) was added to the samples to be used as standard. Then, samples were reconstituted in 100 µL of mobile phase A nanoHPLC-MS analysis performed.

Chromatographic conditions for MS: Mobile phase A, 60/40 (v/v) ACN/H_2O; Mobile phase B, 90/10 (v/v) IPA/ACN. Both modified with 10 mM ammonium formate and 0.1% formic acid. An isocratic method of 40/60 (v/v) mobile phase A/mobile phase B was used, with a duration of 18 minutes.

Mass spectrometer used: Orbitrap Exploris™ 120 (Thermo Fisher Scientific) coupled with an HPLC Ultimate 3000 RSLCnano System (Thermo Scientific). Utilized parameters: Ion transfer tube temperature: 200°C; Spray voltage (positive): 2500 V. Monitored mass range in full scan: 200–600 m/z; Resolution: 60,000. Peak analysis: 369.3516 m/z for endogenous cholesterol and 375.3892 m/z for standard deuterium-labeled cholesterol.

Fluorometric analysis for cholesterol detection: We used the Filipin III as probe for unesterified sterol labeling (Ca. #ab133116, Abcam), following the manufacturer's instructions. The images were acquired via DMi8 Live cell microscope (Leica).

In vivo measurement: Total, LDL, and HDL cholesterol concentration (mg/dL) in animal serum were determined enzymatically using the LabAssay™ Cholesterol (Ca. #293-93601), LDL- Cholesterol (Ca. #291-96701), and HDL-Cholesterol (Ca. #299-96501) kits (FUJIFILM Wako Pure Chemical Corporation) according to the manufacturer's instructions. Serum samples were appropriately diluted in PBS to fall within the linear range of the standard calibration curve. Standards, samples and blank were measured in technical duplicates.

For total cholesterol quantification, 2µL of each sample were mixed with 300µL of chromogenic reagent and incubated at 37°C for 5 minutes. Absorbance was measured at 600nm using the GloMax® (Promega) plate reader.

For LDL and HDL measurements, 180µL of the selective pretreatment reagent were mixed with 5µL of each sample and incubated at 37 °C for 10 min. Then, 90µL of chromogenic reagent were added and incubated again at 37 °C for 10 min before measuring absorbance at 600 nm.

Flow cytometry

Blood samples were treated with ACK lysis buffer for 10 min at 4 °C. The resulting cell suspension was stained with Fixable Aqua Dead Cell Stain Kit (Invitrogen). Cell surface markers were stained

for 20 min at room temperature with the following antibodies: CD45-BV605 (Ca. #103155, Biolegend), CD11b-BUV395 (Ca. #563553, BD Bioscience), CD3-PE-Cy (Ca. #25-0031-82, Invitrogen), CD4-BUV496 (Ca. #564667, BD Bioscience), CD8-BUV805 (Ca. #564920, BD Bioscience), CD49b-BV711 (Ca. #740704, BD Bioscience), Ly-6C-APC-Cy7 (Ca. #560596, BD Bioscience), and Ly-6G-BUV563 (Ca. #612921, BD Bioscience). Cells were fixed and permeabilized with the FoxP3 Transcription Factor Buffer Kit and stained intracellularly with FoxP3-PE-Cy5.5 antibody (Ca. #35-5773-82, eBioscience). Samples were acquired at FACSymphony cytometer (BD Bioscience) and the data were analyzed with FlowJo (v.10.10.0).

In situ hybridization

To detect and visualize the spatial localization of miR-146a-5p in human atherosclerotic plaque, we performed *in situ* hybridization with miRCURY LNA miRNA ISH Optimization Kit (Ca. #339450, Qiagen) on paraffin-embedded tissues, following the manufacturer's indications. Briefly, slides were deparaffinized in xylene and ethanol solutions incubations. Demasking of the miRNAs was done by incubating the tissues for 20 min at 37 °C with Proteinase K to allow the access of the probes to hybridize the miRNAs sequences. After washes with PBS, endogenous peroxidase blockage was performed by incubated tissues with 2% H_2O_2 for 20 min at room temperature. After, the tissues were dehydrated in different passages in ethanol from 70 % to 99.9 % and air-dried for 10 min. Afterwards, tissues were incubated with the double-DIG LNA miRNAs probes, for miR-146a-5p (Ca. #339111-YD00615303-BCG, Qiagen) or a scrambled control (Ca. #90-001, Qiagen), at 40 nM overnight at 53 °C. The next day, SSC washes were performed. Then, tissues were incubated with the blocking solution (1X PBS with 0.1% Tween, 2% sheep serum and 1% BSA) for 30 min at room temperature. After blocking, tissues were incubated for 3 h at room temperature with Anti-Digoxigenin-AP, Fab fragments (Ca. #11093274910, Roche) diluted 1:500 in antibody diluent solution (1X PBS with 0.05 % Tween, 1 % sheep serum and 1 % BSA). After washes with PBS-T 0.1 %, the development of the signal was performed with AP substrate (NBT-BCIP tablet dissolved in Milli-Q water with Levamisol to a final concentration of 0.2 mM) by incubating the slides overnight at 30 °C in a humidifying

chamber. KTBT buffer was used to stop the reaction and two washes in distilled water were performed. After, tissues were incubated with ACTA-2 antibody (Ca. #A5228, Sigma) at 1:5000 dilution for 1 h at room temperature and, after washes, secondary reaction was performed by incubating tissues with MACH1 HRP-polymer (Ca. #M1U539, Biocare Medical) for 15 min at RT. After, DAB (Ca. #DB801, BioCare Medicals) development was performed and finally, tissues were dehydrated and mounted in Eukitt® mountant (Ca. #03989, Sigma-Aldrich).

Small RNA-Seq

All procedures have been performed according to the SMARTer smRNA-seq Kit manufacturer's protocol (Ca. # 635029, Takara Bio). Briefly, to facilitate Oligo(dT)-primed cDNA synthesis, an artificial Poly(A) tail was added, starting from 4 ng of RNA for each sample. After 5 min of incubation with Poly(A) Polymerase and Polyadenylation Master Mix at 16 °C, the samples were ready for cDNA synthesis. The cDNA synthesis step was essential for the addition of adapters to 5' and 3' ends of the first-strand cDNA. For this protocol, samples were maintained at 4 °C for 1 minute before adding 1 μl of 3' smRNAdT Primer to each tube. After that, samples were incubated in a preheated thermal cycler at 72 °C for 3 min, then immediately transferred at 4 °C for 2 min. At this point, Reverse Transcription Master Mix was added to the samples, while keeping them on a PCR chiller rack, and the reaction was run in a thermal cycler with the following program:

**Temperature**	**Time**	**Number of cycles**
42 °C	60 min	1
70 °C	10 min	1
4 °C	Hold	∞

The result of the reaction was a strand of cDNA that had to be amplified, and, at the same time, Illumina adapters had to be added. For this purpose, a PCR reaction was set up using SeqAmp DNA Polymerase, 2X SeqAmp PCR Buffer, forward and reverse primer supplied in the KIT. The reaction was run in a thermal cycler with the following program:

Temperature	Time	Number of cycles
98 °C	1 min	1
98 °C	10 sec	16
60 °C	5 sec	
68 °C	10 sec	
4 °C	Hold	∞

PCR products were purified using the NucleoSpin Gel and PCR Clean-Up kit, according to the manufacturer's protocol (Ca. # 740609.50, Macherey-Nagel), before proceeding with library validation. For library validation, the libraries were quantified on a QUBITFluorometer using the QubitdsDNA HS Assay Kit and then Analyzed through 4200 TapeStation System using Agilent High Sensitivity D5000ScreenTape System. Once the integrity and the good quality of the RNA was established, we proceeded with the size selection of inserts < 150 bp using AMPureXP Beads. Each library was diluted to 4 nM in Resuspension Buffer (Illumina). All libraries were pooled at a concentration of 4 nM before denaturing with 0.2 N NaOH for 5 min at room temperature. The libraries were neutralized using 0.2 N Tris-HCl, pH 7 before being diluted to 20 pM in HT1 buffer (Illumina). Finally, denatured libraries were loaded into an Illumina Next-Seq 500 v2 High-Output reagent cartridge at a final concentration of 1.4 pM in HT1 buffer. Denatured PhiX control library was also included at 5% concentration. Dual indexes and SE75bp sequencing was performed on a High-Output flow cell yielding about 5 million read pairs per sample.

Bioinformatics analysis

miRNA-seq analysis: Single-end multiplexed libraries were sequenced using an in-house NextSeq 500 instrument (Illumina, San Diego, US). A total of 3.7-6.7 million reads per sample were obtained. Adapters from the 3′ end were clipped using cutadapt v2.10, and reads shorter than 15 nucleotides were discarded after adapter trimming. The reads were aligned to *Mus musculus* mature and precursor

miRNAs obtained from miRBase v22 using STAR v2.7.11a. Read counts for miRNAs were quantified using featureCounts v2.0.6, based on the GTF annotation file from miRBase v22. Raw read counts were normalized using TMM, implemented in the edgeR package in R. Lowly expressed miRNAs were filtered out using the filterByExpr function from the edgeR package.

Differential expression analysis was performed using the voomWithQualityWeights() function from the limma package. To specifically evaluate the interaction between cell type (VSMCs vs. Mϕs) and condition (CC vs. MC), custom contrasts were designed to identify miRNAs exhibiting differential expression patterns driven by the interaction effect. This approach allowed us to detect miRNAs potentially transferred from Mϕs to VSMCs.

RNAbulk data analysis: Poly-A sequencing was performed by Novogene Europe. The reads obtained from sequencing were mapped to the GRCm39 genome using STAR aligner v2.7.11b and counted with the Rsubread v2.16.1 package. Genes with fewer than 10 counts in at least one sample per group were removed and the svaseq function of the sva v3.48.0 package was used to identify and remove non-biological variance between replicates; then we used the limma v3.56.2 package with the voom function to prepare the counts to be fitted to a linear model and proceeded to call the differential genes between the 146aOE and the SCRs. Genes with logFC > |1| and with adj.P.Val < 0.05 were selected as differential genes. The clusterProfiler 4.8.3 package was then used to carry out a gene set enrichment analysis (GSEA) and obtain the GOs enriched in the 146aOE with p.adjust < 0.05 and NES > 1. The normalized count matrices with the vst method were crossed with information from the human metabolic model, after the mouse genes have been converted into human orthologs by the biomaRt v2.56.1 package, by the METAFlux v0.0.0.9000 package calculating the metabolic reaction activity scores for each metabolite and the possible flux values of metabolic pathways for human cells cultured in the reference medium available in the package.

Data availability: The gene expression profile dataset is available from the Gene Expression Omnibus (GEO) database (http://www.ncbi.nih.gov/geo/), under the accession number GSE280653. The authors declare that all data supporting the findings of this study are available within the article and its supplementary information files.

ChIP-seq analysis: We obtained the raw data for the ChIP-seq analysis of the GSE112417 project from the Gene Expression Omnibus (GEO) database, using the sample VSMC_Input_1, VSMC_Input_2 and VSMC_SRF. Quality control of the ChIP-seq data was conducted using FastQC. The reads were then mapped to the mm9 genome using the BWA algorithm. Duplicates and unmapped reads were removed with Samtools. Peak calling was performed by MACS2 (version 2.2.8) using the narrow mode and filtered with a p-value threshold of $1.0e^{-6}$. VSMC and the control input samples reads were aligned, filtered, and deduplicated individually. To determine if a gene was regulated by SRF, we considered only the peaks located within a window of 2500 bases upstream of the transcription start site (TSS) and 1000 bases downstream.

Haematoxylin-eosin (H&E)

Slides were deparaffinized and rehydrated before being submerged in hematoxylin for 15 min. After rinsing with tap water, the slides were moved in eosin for 7 min. Finally, after a rapid passage in tap water and in graded alcohols for dehydration, slides were mounted in Eukitt mounting medium.

Immunohistochemistry

Human samples: All immunohistochemical experiments were performed on paraffin-embedded serial slides. Sample slides were deparaffinized and rehydrated. ACTA2 antibody (Ca. #A5228, Sigma-Aldrich) required an unmasking in pressure cooker with slides submerged into DIVA Decloaker buffer (Ca. #BRI2006L, Biocare Medical), while CD68 antibody (Ca. #M0876, Dako) did not need the unmasking. Afterwards, the slides were submerged into 2 % peroxidase solution for 20 min. After being blocked by Background Sniper (Ca. #BS966L, Biocare Medical) for 15 minutes, the slides were incubated with the primary antibody in a humidifying chamber for 1 h at room temperature (1:1000 dilution for ACTA2; 1:100 dilution for CD68). Subsequently, slides were washed with PBS 0.05 % Tween and incubated with MACH-1 Universal HRP-Polymer Detection (Ca. #M1U539, Biocare Medical). After two washes in PBS 0.05 % Tween, we proceeded with the development of the colorimetric reaction using DAB Chromogen Kit (Ca. #DB801, Biocare Medical). After that,

hematoxylin was used to counterstain and, finally, dehydration of the tissues was done, and slides were mounted in Eukitt mounting medium. Negative controls were prepared with the omission of the primary antibody.

Mouse samples: For lipid and fat staining on frozen sections of mouse aortic arch, we fixed slides with 4 % PFA for 15 min at room temperature. Then, the sections were rinsed in 3 changes of distilled water and dried for a few minutes before being immersed in absolute propylene glycol for 5 min. The slides were stained in a pre-warmed Red-oil solution for 10 min at 60 ºC. After Red-oil staining, sections were quickly differentiated in 85 % propylene glycol solution, rinsed in 2 changes of distilled water, and counterstained with Mayer's hematoxylin. Following that, the slides were rinsed in distilled water and mounted with glycerol gelatin aqueous slide mounting medium (Ca. #GG1, Merck). For immunohistochemical experiments F4/80 (Ca. #MCA497G, Bio-Rad), CD3 (Ca. #14-0032-82, eBioscience), ACTA2 (Ca. #A5228, Sigma-Aldrich) antibodies were used. For the quantitative immunohistochemistry experiments, images were acquired using Slide Scanner VS120 dotSlide and analyzed using Image pro premier 9.2 and Image J software.

Circulating proteome profiling and analysis

The analysis was conducted by using the Proximity Extension Assay Olink Target 96 Mouse Exploratory and the Target 48 Mouse cytokine panel (respectively, Ca. #95380 and #934001, Olink Proteomics, Uppsala, Sweden). The Target 96 Mouse Exploratory panel provides protein levels quantification on a relative scale and expressed as normalized protein expression (NPX) units in log2 scale, where higher NPX values indicate higher protein concentrations (Supplementary Table 5), while the Target 48 Mouse cytokine panel allows absolute quantification (pg/mL) for the selected cytokines (Supplementary Table 6). Samples that did not pass the assay quality control are not included in the analysis: final data include 18 animals (9 for DSCR and 9 for D146) and 92 secreted proteins for the inflammation response while 40 cytokines for the cytokine panel. This approach uses specific antibody probes marked with dual oligonucleotides bind to target proteins. Quantitative DNA detection follows, where the oligonucleotide sequence is amplified via microfluidic real-time PCR.

Quality control procedures and normalization were performed on cycle threshold (Ct) data from both internal and external controls.

Supplementary references

1. Farina FM, Hall IF, Serio S, Zani S, Climent M, Salvarani N, Carullo P, Civilini E, Condorelli G, Elia L, Quintavalle M. miR-128-3p Is a Novel Regulator of Vascular Smooth Muscle Cell Phenotypic Switch and Vascular Diseases. *Circ Res* 2020;**126**:e120-e135.
2. Li L, Miano JM, Mercer B, Olson EN. Expression of the SM22alpha promoter in transgenic mice provides evidence for distinct transcriptional regulatory programs in vascular and visceral smooth muscle cells. *J Cell Biol* 1996;**132**:849-859.
